# Magnesium, Zinc and Copper in Lung Fibrosis: A Narrative Review

**DOI:** 10.3390/medicina62010010

**Published:** 2025-12-19

**Authors:** Mihai Nechifor, Carmen Lacramioara Zamfir, Cristina Gales

**Affiliations:** 1 Department of Pharmacology, “Grigore T Popa” University of Medicine and Pharmacy, 700115 Iasi, Romania; mihainechif@yahoo.com; 2 Department of Histology, “Grigore T Popa” University of Medicine and Pharmacy, 700115 Iasi, Romania; carmen.zamfir@umfiasi.ro

**Keywords:** idiopathic pulmonary fibrosis, magnesium, zinc copper

## Abstract

Idiopathic pulmonary fibrosis (IPF) is a chronic lung disease with progressive evolution and high mortality. Magnesium, copper and zinc are essential biometals involved in numerous biological processes in all organs of the human body. A lower level of zinc and magnesium and a higher cooper/zinc ratio are frequently encountered in patients with idiopathic pulmonary fibrosis but also in other forms of pulmonary fibrosis. These imbalances are involved in the main pathogenic mechanisms of idiopathic pulmonary fibrosis: alveolar epithelial cell lesions, oxidative stress, inflammation, fibroblast and myofibroblast proliferation, mitochondrial activity, excessive extracellular matrix accumulation, high collagen production, alveolar macrophage dysfunctions, and apoptosis. A multitude of experimental and clinical studies have shown the importance of these bivalent cations for the synthesis or activity of some important endogenous active substances (fatty acids, eicosanoids, sirtuin1, p53 protein, interleukins, growth factors, some enzymes, and others) involved in one form or another in the pathogenesis of IPF. There are no randomized clinical trials yet, but some clinical and experimental results suggest that the association of zinc and magnesium with pirfenidone and nintedanib could be beneficial and should be assessed as soon as possible after the onset of this disease. The correction of hypomagnesemia and hypozincemia, whenever they exist, must be performed as soon as possible after the diagnosis of fibrosis.

## 1. Introduction

Pulmonary fibrosis is one of the most severe pathological states of the lungs. In the case of both idiopathic pulmonary fibrosis (IPF) and fibrosis developed as a complication of other diseases or through the action of some harmful agents, the impairment of pulmonary function is important and the therapeutic solutions are few. Pulmonary fibrosis is a chronic progressive disease that greatly disrupts lung functions, reduces gas exchange, causes disorganization of the alveolar architecture, and significantly reduces pulmonary compliance [1]. The result of all these pathological changes is, sooner or later, the death of the patient. The evolution of pulmonary fibrosis is aggravated by aging. For pulmonary fibrosis, as for other diseases, aging is an additional risk factor [2,3]. Worldwide, there are about 3–5 million patients with IPF and many others with fibrosis secondary to the action of various known pathogenic factors [4]. Most cases of IPF are diagnosed in people over the age of 60. In these cases, the median survival of patients after diagnosis is about 3–5 years [5,6]. Some studies show an incidence of IPF of 400 per 100,000 people over the age of 65 [7]. According to ATS data, the incidence of IPF increases with age [8]. Unfortunately, therapeutic solutions for this serious disease are few. The most promising drugs currently used are pirfenidone and nintedanib [9,10]. The same thing happens with the incidence of magnesium and zinc deficiency.

Another large number of other patients have different degrees of pulmonary fibrosis in diseases such as diabetes [11,12], post-irradiation, in cases of silicosis and anthracosis, in smokers, patients with chronic pulmonary infections [13,14] and in other people. IPF has a complex pathogenesis that is still incompletely known despite all the great progress made in this direction in recent years. A serious and still incompletely elucidated problem is the relationship between IPF and the development of lung cancer. Some studies show that about 22% of patients with pulmonary fibrosis develop lung cancer, and the risk for lung cancer is five times higher in patients with IPF than in the general population [15,16].

Magnesium, zinc and copper are three of the bivalent biometals in the human body. All three are essential bioelements in our body. Magnesium is required for good mitochondrial function, is majorly involved in the regulation of the release of synaptic mediators, has implications in the stability of DNA and RNA, reduces oxidative stress, and has anti-inflammatory, antidepressant, anxiolytic, and antipsychotic action and many other functions in the human body [17]. The minimum normal value of serum magnesium in healthy adults is considered to be 0.85 mmol/L (1.7 mEq/L or 27 mg/L) [18,19]. A significant number of people from all over the world have chronic asymptomatic hypomagnesemia with 0.6–0.85 mmol/L magnesium. Magnesium is a cation located mainly intracellularly. This constitutes about 98% of the total amount of magnesium in the body. In the cells of mammals, the concentration of magnesium varies between 15 and 18 mmol/L. The cell organelles richest in magnesium are the mitochondria, the endoplasmic reticulum, and the nucleus. Aging is associated with a decrease in magnesium concentration [20]. After 60 years of age, the incidence of pulmonary fibrosis increases significantly [21].

Copper is a very important trace element in the human body. This biometal is located both intracellularly and extracellularly [22]. The concentration of copper in the human body varies depending on the organ, age, and state of health. In healthy people aged 14–80 years, the concentration of this biometal in the lungs is 1.91 ± 1.30 micrograms of copper/g. The serum concentration of copper in healthy adult subjects is 0.85 ± 0.19 micrograms of copper/mL in some studies [23] or more in other studies [24]. Some studies showed that the concentration of serum copper of healthy people was 14.5 + 1.92 µmol/L [25]. In the body of a healthy adult of average weight, there is between 100 and 200 mg of copper, of which 50–70% is found in the bones and muscles and 20% in the liver. Therefore, the amount of copper in the lungs is small [26]. Copper is involved in more than 30 metalloproteins that are copper-dependent enzymes. This trace element is involved in mitochondrial respiration, the synthesis of active biogenic substances, and energy metabolism. More than 300 enzymes and transcription factors are dependent on zinc for their normal activity. This biometal has essential roles in immunity, male fertility, and reduction of oxidative stress; an anti-inflammatory role in the release of some neuro-mediators; in the functioning of some central synapses; and in cognition and behavior [27,28]. The human body, a healthy adult weighing 70 kg, contains about 2.2 g of zinc. The normal serum concentration of zinc is 19.7 ± 0.24 μmol/L. The incidence of zinc deficiency is high. About 30% of healthy elderly people in developed countries and between 20 and 30% of the entire population of some developing countries have a greater or lesser deficiency in zinc [27,29]. In bronchoalveolar lavage fluid (BALF), the zinc concentration is significantly lower than in normal people [30]. A low serum zinc concentration is associated with an increased risk of lung cancer [31]. An increased serum copper/zinc ratio is also associated with an increased risk of lung cancer [32].

There are several known pathogenic mechanisms of pulmonary fibrosis. The most important pathogenic mechanisms in the pathogenesis of fibrosis are imbalances regarding oxidative stress; inflammation; fibroblast and myofibroblast proliferation; mitochondrial activity; excessive extracellular matrix accumulation and high collagen production; alveolar macrophage dysfunctions; and apoptosis [33]. An important number of active endogenous substances have implications in the pathogenesis of pulmonary fibrosis. Some of the most important are fatty acids, eicosanoids (leukotrienes, prostaglandins, lipoxines, hepoxilines, resolvins), SIRT, protein p53, nitric oxide, some transduction factors and others [34]. The purpose of this review is to highlight the involvement of magnesium, zinc, and copper in the pathogenesis of pulmonary fibrosis. Databases used for this review were PubMed and Google scholar. The search strategy included only full texts. The keywords used for the search were magnesium, zinc, copper, and pulmonary fibrosis. Inclusion criteria were as follows: pulmonary fibrosis diagnosed according to current criteria, experimental fibrosis produced by a validated experimental modality. Exclusion criteria were as follows: only abstract, case reports. Experimental in vivo and in vitro studies and clinical trials were included. No limits were set regarding time.

## 2. Oxidative Stress

An imbalance between prooxidant and antioxidant factors at the pulmonary level is very important in the pathogenesis of IPF. Oxidative stress is definitely an important factor in the development of pulmonary fibrosis. This type of cell aggression significantly increases in patients with this disease compared to healthy people of the same age. The increase in this stress correlates positively with the development of pulmonary fibrosis and negatively with Forced Vital Capacity (FVC) [35]. Reactive oxygen species (ROS) are also involved in the production of alveolar cell damage. In experimental studies, administration of superoxide dismutase eliminated superoxide toxicity and significantly improved the evolution of pulmonary fibrosis [36]. In patients with chronic obstructive pulmonary disease, the plasma level of magnesium is low and oxidative stress is higher than in healthy people [37]. The expression of important antioxidant factors such as Nrf2 (nuclear factor erythroid 2-related factor 2) is low and the formation of free radicals increases significantly [38]. The experimental administration of endotoxin in rats causes a significant increase in oxidative stress and various lung injuries. MgSO4 (50–100 mg/kg) administered to animals significantly reduced oxidative stress and lung lesions associated with it [39]. Inhibition of nuclear factor-kappaB (NF-κB) activity is an important element in reducing pulmonary oxidative stress and inflammation [40]. Magnesium acts by inhibiting signaling on the NF-κB pathway [41], and by inhibition of NF- κB activity, a TLR4/NF-κB signaling pathway in the lung is depressed. Through this mechanism, the synthesis of pro-inflammatory cytokines in lung tissues is reduced [42].

**Figure 1:**
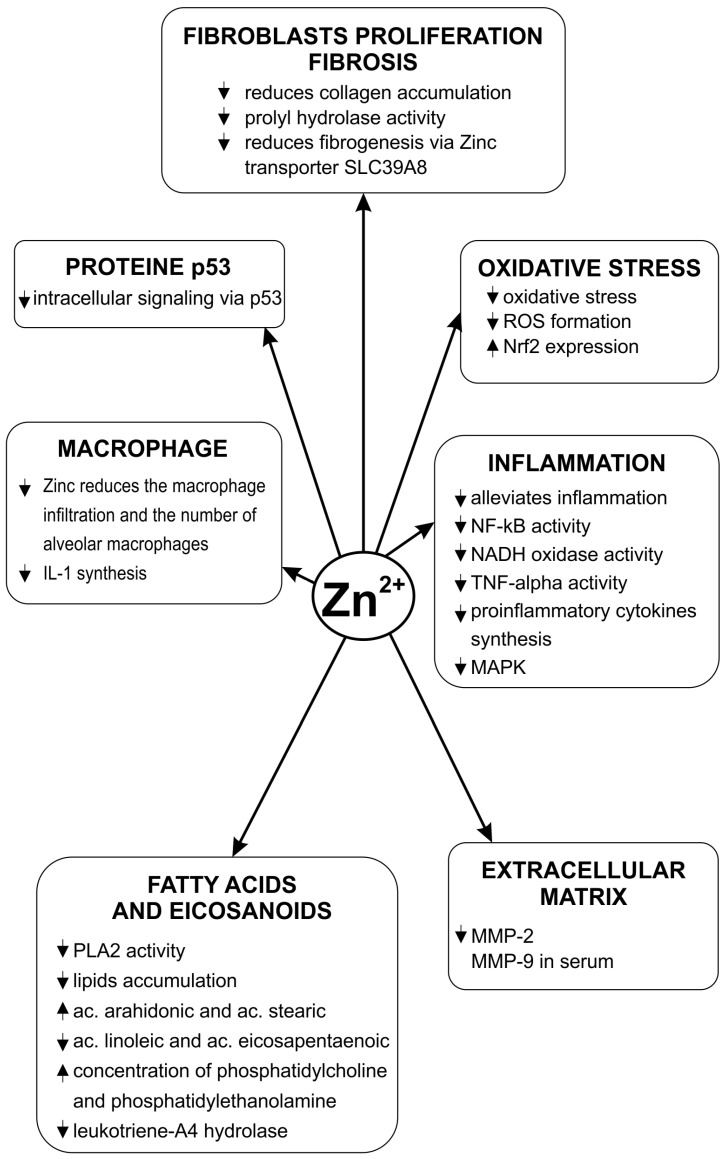
Zinc’s influence on the pathogenic mechanisms of pulmonary fibrosis.

Zinc deficiency exacerbates oxidative stress in the lungs, increases the production of ROS and decreases the expression of Nrf2 [38]. Copper produces a concentration-dependent increase ROS generation [43]. Copper/zinc (Cu-Zn) superoxide dismutase (SOD) is an essential enzyme in the protective mechanisms against oxidative stress. Its normal functioning requires the existence of both bivalent cations and a balance between their concentrations. Dysregulation of reduced glutathione (GSH) synthesis contributes to the pathogenesis of pulmonary fibrosis [44]. The action of various toxic factors on the lung is associated with a decrease in the level of GSH at the pulmonary level. Zinc antagonizes this effect and increases the level of GSH [45]. Some experimental pathological situations, such as intestinal ischemia–reperfusion and acute lung injury, are mediated in part by increased oxidative stress. Administration of 50 mg/kg zinc aspartate to these animals significantly reduces the pulmonary level of malondialdehyde (MDA), nitric oxide, and myeloperoxidase. This compound increases the activity of glutathione peroxidase in both the lung and bronchoalveolar lavage fluid [46]. Zinc sulfate improves pulmonary oxidative stress and increases GSH level in rats with streptozotocin-induced diabetes [47]. Zn deficiency in rats resulted in increased lipid peroxidation and SOD and GSH-Px (glutathione peroxidase) activities [48]. An experimental study showed that administering zinc sulfate in the case of lung injury produced an increase in SOD levels and alleviated lung injury [49]. The involvement of zinc in the pathogenesis of pulmonary fibrosis is shown in Figure 1.

Copper concentration is also important for oxidative stress. The increase in Cu concentration in the lungs is associated with increased oxidative stress in patients with cystic fibrosis [50].

In an experimental study of copper deficiency, plasma GSH concentration was significantly increased. The copper(II) catalyzed GSH oxidation [51].

## 3. Inflammation

Pulmonary inflammation is an essential process in the pathogenesis of idiopathic pulmonary fibrosis. This disease has among its key pathogenic elements an increase in the number and activity of pro-inflammatory leukocytes in the lungs [52]. An important number of pro-inflammatory cytokines, growth factors and transcription factors are involved in the pathogenesis of IPF. Transforming-growth factor beta 1 (TGF-β1) and the pro-inflammatory interleukins Il-1β, IL-4, IL-13, and Il-18 are involved in stimulating the formation of pulmonary fibrosis [53]. An increase in NF-κB signaling pathway activity determines the excess synthesis of some pro-inflammatory factors such as pro-inflammatory cytokines. Inhibiting the NF-κB/NLRP3 signaling pathway reduces inflammation and the development of pulmonary fibrosis [54]. Zinc has anti-inflammatory actions [55]. Zinc also reduces the activity of the transcription factor NF-κB. An increased concentration of this micronutrient is associated with elevated activity of pro-inflammatory factors. The zinc transporter ZIP8 regulates host defense through zinc-mediated inhibition of NF-κB [56].

Experimental studies have shown that zinc deficiency increases inflammation and favors the development of pulmonary fibrosis in mice [38]. Zinc alleviates inflammation induced by lipopolysaccharide (LPS) and other pro-inflammatory factors [56]. A diet deficient in zinc in rats produced an increase in activity in some pro-inflammatory factors such as NADH oxidase, tumor necrosis factor alpha (TNF-α), and others. Supplementing the diet with zinc even for a period of 10–14 days causes a reduction in the synthesis and activity of these pro-inflammatory factors at the pulmonary level [57]. There is data showing that magnesium isoglycyrrhizinate (MgIG) reduces the formation of pro-inflammatory factors determined by LPS. Reducing magnesium concentration activates TNF-α/NF-κB signaling, which leads to intensification of inflammation. MgIG (0.80 mg/kg/day) significantly reduced the concentration of IL-6 and TNF-α at the lung and serum levels in rats with experimentally induced chronic obstructive pulmonary disease COPD [58]. This compound inhibits the development of inflammation and fibrosis by inhibiting the signaling pathway related to NF-kB [59]. A low magnesium concentration causes an increase in the expression of p38-mitogen-activated protein kinase (MAPK) at the pulmonary level [60].

In a double-blinded, placebo-controlled trial, administration of 250 mg/day magnesium oxide for 6 weeks downregulated gene expression levels of interleukin-8 (c) and TNF-α [61]. C-reactive protein (CRP) levels were significantly higher in IPF patients than normal controls [62]. Some authors considered this protein as a biomarker in IPF evolution [63].

Magnesium citrate 300 mg/day in patients with COPD significantly reduced the level of CRP [57,64]. In the case of a diet low in copper, an accumulation of neutrophils occurs in the lungs [58,65]. Contrary to zinc and magnesium, copper activates the NF-kB signaling pathway in the lung and in other organs [66,67].

The MAPK (mitogen-activated protein kinase) pathway is the activation pathway of the extracellular regulated protein kinases ERK1 and ERK2. ERK1/2 are enzymes involved in numerous normal and pathological processes throughout the body, including at the lung level. Among the processes in which these enzymes are involved, we mention the proliferation of progenitor cells and apoptotic cell death. Both enzymes are involved in the phosphorylation of a large number of proteins. Magnesium is one of the important elements in ERK activation. A concentration of 2 mmol/L Mg^2+^ increased the phosphorylation of ERK1 and ERK2 by about two times [68]. ERK2 must bind two divalent magnesium ions. The administration of magnesium in cell culture increased the activity of MAPK, ERK 1, and ERK 2 [69]. Magnesium Isoglycyrrhizinate (MgIG) reduces inflammation by blocking the NF-κB and MAPK signaling pathways [70].

In cell cultures, a decrease in zinc concentration is associated with a reduction in cell proliferation and the blocking of the cell cycle at the G0/G1 phase [71]. The main way through which zinc regulates biological signal transduction mediated by ERK1/2 is the inhibition of phosphatases that determine the dephosphorylation of ERK1 and ERK2. Zinc-sensing receptors on the cell membrane are also very important. Zinc-sensing receptor desensitization causes a strong reduction in ERK1 and ERK2 phosphorylation [72]. On the other hand, in some forms of lung cancer, the transcription factor ZNF424 (which is dependent on zinc) inhibited cell proliferation and reduced the synthesis of proteins involved in the activity of the transduction pathway mediated by MAPK [73]. MAPK plays a role in Cu-induced premature cell senescence in the lung [74].

Sprague Dawley rats that received 250 ppm of copper daily in drinking water for 16 weeks showed an increase in oxidative stress and pro-inflammatory factors at the lung level, simultaneously with an intensification of copper accumulation in the lungs [75]. Excess copper, as well as a copper/zinc ratio higher than normal, has a pro-inflammatory action [76].

## 4. Mitochondrial Dysfunctions

Mitochondrial dysfunctions have been identified at the level of the main cells (fibroblasts, alveolar cells, macrophages) involved in the pathogenesis of IPF. The production of ROS at the mitochondrial level is increased, and the energy metabolism is disturbed [77]. This increased mitochondrial synthesis of ROS is important in the genesis of pulmonary fibrosis because in experimental models, it was shown that mice that overexpress mitochondria-targeted catalase (a ROS scavenger) show partial protective effects against the development of pulmonary fibrosis [78]. The level of mitochondrial ATP in lung fibroblasts is reduced, and cellular energy production is also low in pulmonary fibrosis. In alveolar macrophages, fatty acid oxidation is increased [77]. Magnesium is essential for the activity of ATP and the production of energy. In cells, ATP is found in a form linked to magnesium. A low concentration of intracellular magnesium is associated with less energy production. Determining the intracellular concentration of magnesium, which unfortunately is rarely performed in clinical practice, is important for all patients with IPF, and any deficiencies must be immediately corrected by administering magnesium compounds. Copper activates oxidative stress, and in concentrations higher than normal, it causes mitochondrial dysfunction. Copper also induces an increased deposition of lipids [79]. This biometal produces swelling and decreases the mitochondrial membrane potential [80].

## 5. Excessive Extracellular Matrix Accumulation

Matrix metalloproteases (MMPs) have a major role in regulating the amount of extracellular matrix. They are endopeptidases capable of degrading all components of the extracellular matrix.

Their inhibition increases the severity of pulmonary fibrosis and increases the amount of extracellular matrix in the lung. Bivalent cations have a different influence on these enzymes.

MMPs have a double entanglement in idiopathic pulmonary fibrosis. They increase alveolar permeability, but on the other hand, it has been shown in different types of experimental fibrosis that their inhibition worsens fibrosis [81]. The level of these enzymes is higher in IPF than in healthy people. Bivalent cations have a different influence on these enzymes. Magnesium isoglycyrrhizinate inhibited the activity of MMP-9 in rats with lung injury induced by paraquat poisoning [82].

In human cell cultures, magnesium concentrations of 0–3.0 mmol/L significantly reduced the production of MMP2 stimulated by homocysteine [83]. In patients with chronic kidney disease, serum zinc levels are negatively correlated with the concentration of MMP-2 and MMP-9 [84]. In experimental studies on rabbits, the addition of zinc to a normal diet or to a high-fat diet significantly reduced MMP2 and MMP9 expression [85]. Data regarding the influence of copper on MMPs are contradictory. Administration of glycyl-L-histidyl-L-lysine-Cu(II) in rats activated MMP2 during the final stages of healing lesions [86]. Copper–methionine administration in cold-stressed broilers decreased the concentration of MMP-2 [87].

## 6. Fibroblast and Myofibroblast Differentiation and Fibrosis

Fibroblast and myofibroblast proliferation is the essential pathogenic mechanism of IPF [88]. A lot of factors are involved in the development of pulmonary fibrosis; some authors believe that TGF-β1 plays the main role. In the alveolar epithelial cells (AECs) and pulmonary macrophages of patients with IPF, the concentration of TGF-β is greatly increased. This factor initiates fibrosis and is essential for the changes at the level of lung fibroblasts and the extracellular matrix [89]. ROS stimulate TGF-β1 synthesis by epithelial alveolar cells [90]. Besides its own profibrotic action, TGF-β1 activates the synthesis of other pro-inflammatory cytokines that are involved in the development of pulmonary fibrosis, such as IL-1β and TNF-α [91]. This increase was demonstrated both in patients with IPF and in the case of animals in which pulmonary fibrosis was induced with bleomycin [92]. TGF-β3 also plays a role in pulmonary fibrosis development. This factor reduces procollagen degradation [93]. Other profibrotic factors involved in the development of IPF are platelet-derived growth factor (PDGF) and basic-fibroblast growth factor (bFGF [94]. Some heavy metals, such as manganese and lead, induce pulmonary fibrosis by activating extracellular signal-regulated kinase (ERK) in pulmonary fibroblasts [95]. Of course, the translation of experimental results obtained in experimental animals into the human clinic always carries some risks. In the case of experimental pulmonary fibrosis, there are mechanisms similar or close to those in human pathology. One of the differences between experimental fibrosis and human clinical pulmonary fibrosis is that experimental fibrosis occurs in an incomparably shorter time than human pulmonary fibrosis, which develops over several years.

An Mg-deficient diet in rats induced a 19% increase in collagen synthesis and deposition [96]. The reduction of the expression of Toll-like receptor (TLR) 4 and NF-κB by various harmful factors is one of the mechanisms of the development of fibrosis [59]. Toll-like receptors (TLRs) also play a role in pain reception, in immunity and in the functioning of ion channels. Magnesium does not enter cells through passive diffusion, but through some proteins with ion channels as well as through the activity of an enzyme called transient receptor potential melastatin (TRPM) and with the help of some transporters. TRPM channels consist of a channel protein linked to a cytosolic protein kinase. Among these TRPMs, the most important at the level of current knowledge are TRPM6 and TRPM 7. Among the members of this enzyme-channel superfamily, there are data showing that TRPM 7 is involved in the development of fibrosis in different organs, including pulmonary fibrosis. It is considered that the mechanism by which TRPM 7 is involved in the pathogenesis of pulmonary fibrosis is stimulation of the activity of transforming growth factor-β1 (TGF-β1) at the level of human pulmonary fibroblasts [97]. The activation of TGF-β1 determines the stimulation of fibroblast-to-myofibroblast transition and the increase in collagen and elastin production. It is considered that the mechanism by which TRPM 7 is involved in the pathogenesis of pulmonary fibrosis is the stimulating the activity of TGF-β1 at the level of human pulmonary fibroblasts. The activation of TGF-β1 determines the stimulation of fibroblast-to-myofibroblast transition and the increase in collagen and elastin production [98,99]. The antifibrotic action of magnesium lithospermate B (MLB) in experimental fibrosis induced with bleomycin is produced by inhibiting transforming growth factor-beta (TGF-β)-stimulated myofibroblastic transdifferentiation of lung fibroblasts [100]. MgIG also reduced the pulmonary concentration of TGF-1 beta in radiation-induced experimental pulmonary fibrosis and decreased fibrosis progression [99].

**Figure 2:**
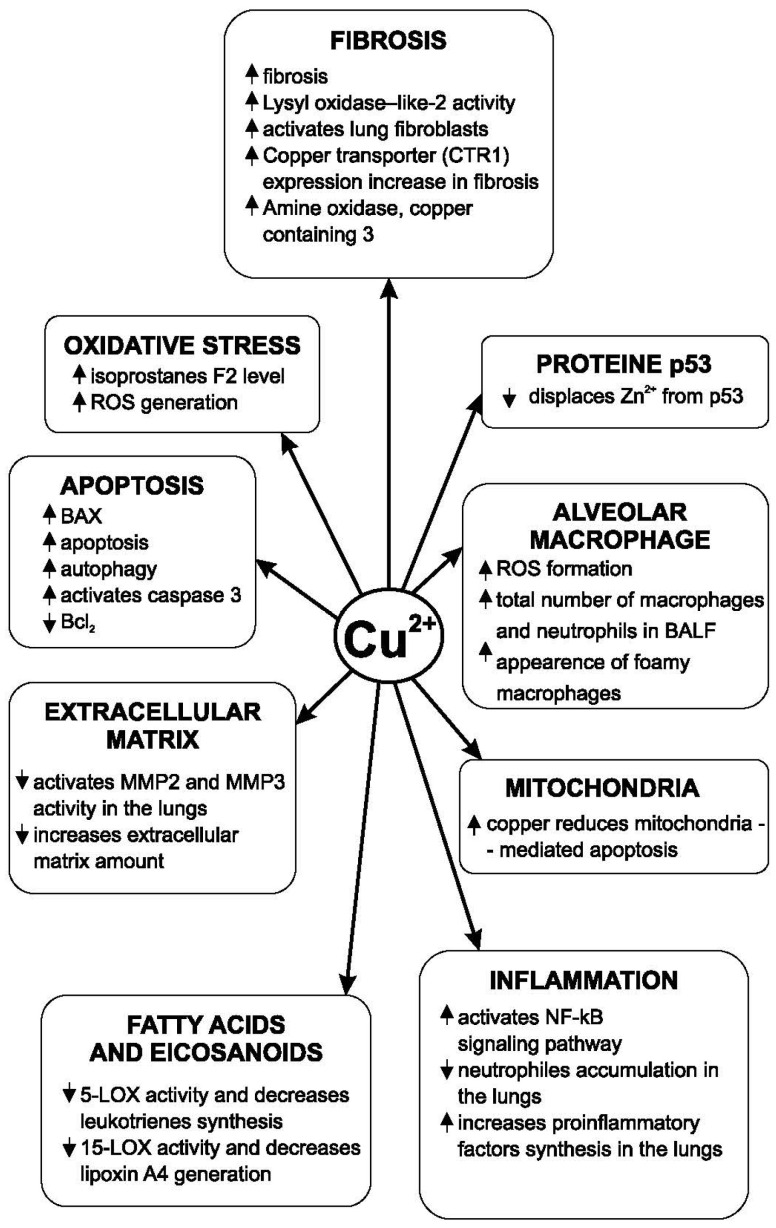
Copper’s influence on the pathogenic mechanisms of pulmonary fibrosis.

A 114 mg/L amount of zinc in drinking water reduced collagen accumulation in the lungs of animals experimentally intoxicated with CCl_4_. Zinc’s mechanism of action is the inhibition of the lung prolyl hydroxylase activity [101]. In lactating rats that received barium chloride, the administration of zinc (50 mg/kg by gavage) reduced the deposition of collagen in the lungs. At the same time, this biometal increased the antioxidant defense [102]. In the case of experimental pulmonary fibrosis induced by viral infections, some authors consider that NF-κB plays an essential role [103]. Copper has an opposite action to zinc and magnesium regarding the development of pulmonary fibrosis. Lysyl oxidase-like 2 (LOXL2) is also involved in the development of lung fibrosis and plays an important role. This enzyme, which activates lung fibroblasts, is a copper-dependent monoamine oxidase [104]. Some data shows that copper is also involved in the development of fibrosis in other organs. Copper transporter 1 (CTR1) expression was increased both in patients with renal fibrosis and in the case of experimental fibrosis. The involvement of copper in the pathogenesis of pulmonary fibrosis is shown in Figure 2.

**Figure 3:**
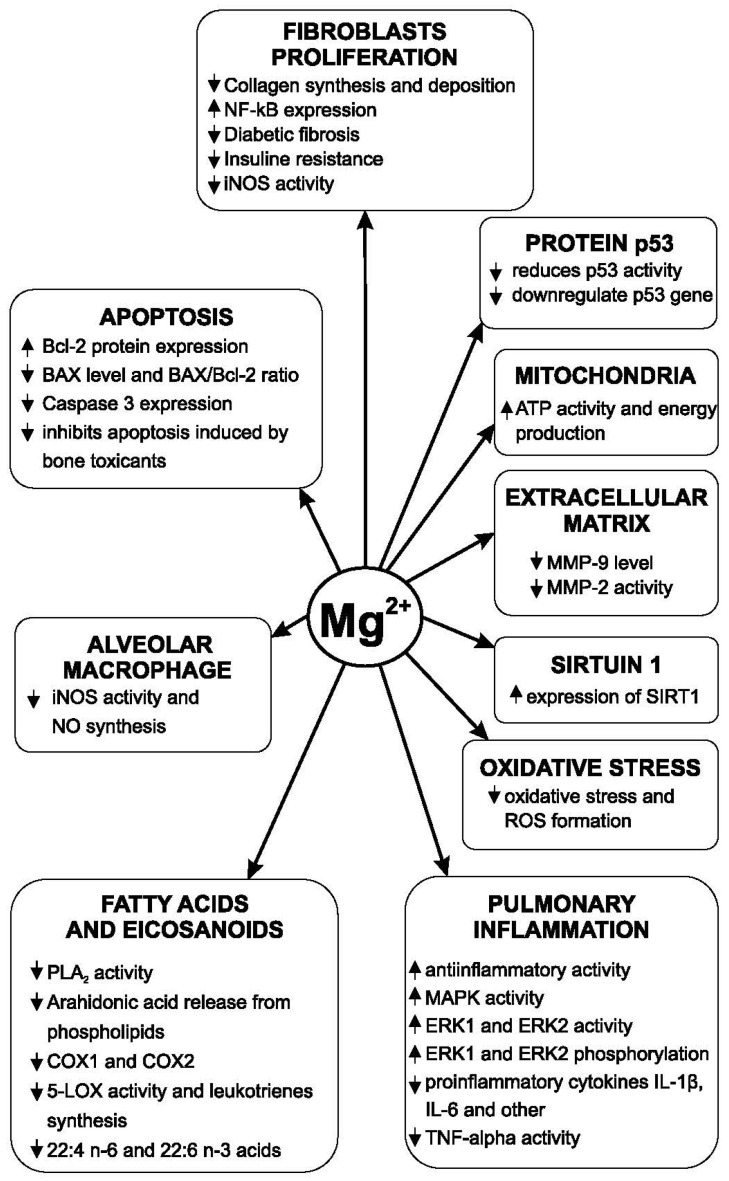
Magnesium’s influence on the pathogenic mechanisms of pulmonary fibrosis.

The main mechanism of action of intracellular copper ions is the activation of LOXL (lysyl oxidase-like) [105]. LOXL is an essential enzyme in collagen synthesis and the formation of the extracellular matrix. This enzyme is copper-dependent. Copper stimulates an increase in the amount of extracellular matrix [106]. The increase in the expression of LOXL is positively correlated with the development of pulmonary fibrosis and the increase in the amount of extracellular matrix in patients with IPF [107]. In the genesis of pulmonary fibrosis, it is important not only to exacerbate the production of collagen fibers but also the accumulation of elastin fibers. After the exacerbation of pulmonary fibrosis, the plasma copper level increases [108]. The LOXL is a copper-dependent enzyme that has a regulatory role in the synthesis and accumulation of elastic fibers in the lungs [109]. Copper ions are also involved in another way in the development of pulmonary fibrosis. Amine oxidase copper-containing-3 (AOC3) is an enzyme with a role in the development of pulmonary fibrosis. Inhibition of this enzyme (or its deficiency) reduced the development of fibrosis and the accumulation of collagen by 30–50% in experimental fibrosis induced with bleomycin in mice [110]. TNF-α is also present at a high level in the supernatant of fibroblast cultures in the case of IPF. Some growth factors, such as fibroblast growth factor (FGF), platelet-derived growth factor (PDGF), and vascular endothelial growth factor (VEGF), are also involved in the progression of pulmonary fibrosis [111]. Tyrosine kinase is involved in cellular stimulation by these growth factors. Inhibition of tyrosine kinase reduces the action of these growth factors [112]. Exposure to CuSO4 (100 μM concentration) in vitro produces a decrease in the viability of both alveolar cells and bronchoepithelial cells. At the same time, this copper compound amplified epithelial–mesenchymal transition and increased cell migration and profibrotic gene expression [113]. Prolonged activity of miners in copper mines with a high concentration of this element leads to the development of an important type of pulmonary fibrosis [114]. Copper also increases autophagy [80] and inhibits acetylcholinesterase [115]. Magnesium lithospermate B (50 mg/kg/day for 7 days) has an antifibrotic effect. The mechanisms by which this magnesium derivative produced its effect are reductions in TGF-β synthesis and activity, decreased expression of receptors for TGF-β1, reduced collagen synthesis, and decreased myofibroblastic transformation of lung fibroblasts [100]. The level of superoxide dismutase (SOD) and glutathione peroxidase (GSH-PX) activities was significantly higher after magnesium lithospermate B administration decreased the MDA concentration. Fibrosis produced by experimental irradiation is also reduced by this magnesium compound. The reduction of the development of pulmonary fibrosis by this compound occurs primarily by inhibiting the differentiation of pulmonary fibroblasts by influencing the MAPK/Akt/Nox4 biological signal transduction pathway. The MAPK pathway is the activation pathway of the extracellular regulated protein kinases ERK1 and ERK2. Both enzymes are involved in the phosphorylation of a large number of proteins [116]. Magnesium reduces Nox4 (NADPH oxidase 4) expression. Nox4 has increased expression at the level of lung fibroblasts in patients with idiopathic pulmonary fibrosis and is essential for the production of the TGF-beta 1 effect at the level of fibroblasts. Phosphorylation of p38MAPK and Akt (protein kinase B) is significantly reduced [58]. The development of pulmonary fibrosis in patients with type 2 diabetes is associated with the constant hypomagnesemia encountered in these patients and with increased insulin resistance. Magnesium increases insulin receptors’ sensitivity to their natural agonist [117]. Nitric oxide is involved in the development of IPF. Bleomycin induces experimental pulmonary fibrosis through multiple mechanisms. One of these is the induction of increased angiogenesis, the activation of VEGF, and the increase in pulmonary synthesis of nitric oxide [118]. VEGF not only stimulates angiogenesis but also increases vascular permeability and cell proliferation. This factor is found in significant quantities in macrophages, lung fibroblasts and alveolar cells [119]. Clinical studies have shown a much higher level of nitric oxide in exhaled air in patients with IPF compared to healthy people [120]. The alveolar concentration of nitric oxide is increased in the alveoli of patients with IPF, and some authors believe that it is correlated with the severity of fibrosis and could be a biomarker for the evolution of this disease [121]. The existing data regarding the action of magnesium on the synthesis of nitric oxide in different organs are heterogeneous. In some studies, a low level of extracellular magnesium causes increased nitric oxide release and upregulated inducible nitric oxide synthase (iNOS) [122]. The activity of iNOS is increased at the level of the lungs in zinc deficiency [123]. Intravenous administration of a solution containing copper causes a decrease in blood pressure in animals and an important increase in iNOS activity [124]. Magnesium’s influence on the pathogenic mechanisms of pulmonary fibrosis is presented in Figure 3.

## 7. Alveolar Macrophages

In the lungs and other organs, two types of macrophages have been identified, called M1 and M2, respectively. M1 macrophages produce pro-inflammatory cytokines and TNF-α. On the other hand, M2 macrophages reduce inflammation and play a role in tissue repair [125]. The role of alveolar macrophages in pulmonary fibrosis is complex because these cells are involved not only in fibrogenesis but also in fibrolysis [126]. There are two different phenotypes of macrophages: one pro-fibrotic and the other fibrinolytic. M2 macrophages are involved in the development of pulmonary fibrosis. They secrete profibrotic cytokines [127]. M2 macrophages play an important role in the development of fibrosis, and inhibiting cells’ activity has a protective effect in the development of experimental pulmonary fibrosis [128]. Some authors consider monocyte-derived alveolar macrophages to be one of the main pathogenic factors in idiopathic pulmonary fibrosis [129]. The decrease in magnesium concentration from normal (0.39 mM to 0.021 mM) in the culture medium of alveolar macrophages determines an increase in the synthesis of nitric oxide through the stimulation of iNOS. In vivo, a decrease in the serum concentration of magnesium also causes an increase in the release of nitric oxide [130]. In experimental models of mice exposed to cigarette smoke, the number of macrophages in bronchoalveolar lavage fluid (BALF) increased twice, but supplementing the diet with zinc reduced the number of alveolar macrophages by 52% and reduced pulmonary inflammation [131]. There are no data regarding a differentiated action of zinc, magnesium, or copper on type M1 and M2 macrophages. Zinc deficiency increased macrophage infiltration [56]. This cation also has a modulatory role in the production of IL-1 alpha in human lung macrophages [132]. During bacterial infections, copper accumulates in the phagosomes of alveolar macrophages [133]. Exposure of alveolar macrophages to copper oxide increases the generation of ROS in these cells [108]. The copper transporter ATP7A was identified in macrophages. This transporter is involved in the modulation of extracellular superoxide dismutase activity [134]. Exposure of animals to Cu SO4 in air produces an increase in the plasma concentration of isoprostane F2 (an important marker of oxidative stress), an increase in the total number of neutrophils and macrophages in BALF and the appearance of foamy macrophages in the lung [135]. Zinc is a potent modulator of pulmonary macrophage function in response to harmful stimuli. The main mechanism by which this effect is produced is mediated by NF-κB and MAPK signaling pathways [136]. The administration of an alcohol-fed diet in rats for 6 weeks induces other dysfunctions of pulmonary macrophages and AECs. These dysfunctions are aggravated by a low level of zinc and partially improved by the administration of zinc [137].

## 8. Alveolar Epithelial Cells

Impairment of the normal activity of alveolar epithelial type II cells is considered a key factor in the pathogenesis of IPF. Some authors consider that type II alveolar epithelial cell (AECII) injury is sufficient to cause pulmonary fibrosis [138].

Deficient repair of alveolar epithelial cell lesions is a major cause of pulmonary fibrosis [139]. AECII cells represent a lung cell population important for surfactant biosynthesis and also for alveolar epithelial regeneration by transdifferentiation into AEC 1 [140,141]. In the pathogenesis of pulmonary fibrosis, the repair action of AECII at the level of alveolar epithelial lesions is insufficient or ineffective [142]. Dysfunctions at the level of AT2 cell telomeres are involved in alveolar progenitor senescence. This prevents efficient repair of alveolar epithelial lesions [143]. Shorter telomeres in idiopathic pulmonary fibrosis are positively associated with the intensity of fibrotic lesions [144]. Increased apoptosis of AECII is important in the pathogenesis of IDF [145]. This apoptosis is very important because intense myofibroblast activity occurs in areas adjacent to areas of intense apoptosis, [146]. Lesions of the alveolar epithelium are multiple because in fibrotic foci, there are not only areas of alveolar denudation but also areas of epithelial hyperplasia. Substances such as bleomycin that induce senescence of alveolar epithelial cells also cause pulmonary fibrosis. Inhibition of AEC senescence by tetrandrine alleviates bleomycin-induced experimental pulmonary fibrosis [147]. One study showed that enhanced expression of IL-17 receptor A in patients with idiopathic pulmonary fibrosis. IL-17 induces mitochondrial dysfunction and stimulates AEC apoptosis. In IL-17A-knockout mice, both AEC apoptosis and bleomycin-induced experimental pulmonary fibrosis are significantly reduced [148]. AECs synthesize some factors such as platelet-derived growth factor (PDGF), transforming growth factor (TGF)-β, and tumor necrosis factor (TNF-α). Their production in excess when there is damage to AECs is an important element in the production of pulmonary fibrosis [149]. TGF-β increases phosphorylation of Smad2/3 (Smad transcription factor 2/3) and activates pulmonary fibrogenesis. Magnesium lithospermate B reduces the action of TGF-β on its receptors, decreases phosphorylation of Smad2/3, and reduces collagen deposition in the lung and fibrogenesis [100]. LPS produces inflammatory reactions, apoptosis, and barrier breakdown in alveolar epithelial cells. Magnesium hydride (MgH_2_) diminishes all these processes, maintaining barrier integrity and reducing the possibilities of stimulating pulmonary fibrosis. MgH_2_ also suppressed barrier breakdown by upregulating the expression of occludin [150].

When different agents damage AECs, a replacement of the destroyed cells by AEC II occurs. Any disruption of this process leads to a reduction in the activity of the barrier between the organism and the external environment and to excess stimulation of fibroblasts by TGF-β and other factors [149]. An important function of AEC2s is in progenitor cells in the lung. Reduction in this function is associated with the development of pulmonary fibrosis. Zinc is required for this function of AEC2s. Deficiency of a zinc transporter, SLC39A8 (ZIP8), has been identified in AEC2s in patients with IPF [151]. Dysfunctions induced by some toxicants such as ethanol at the AEC level are partially mediated by zinc deficiency [137]. In AECs, copper oxide increases oxidative stress and produces morphological changes. Activation of autophagic cell death occurs after internalization of CuO particles. This compound also induces mitochondrial damage [152]. Endoplasmic reticulum stress at the level of the alveolar epithelium is also implicated in the pathogenesis of IPF [13]. Viral infections (e.g., herpes virus infection) that produce endoplasmic reticulum stress of the alveolar epithelium are associated with an increased incidence of IPF [153,154].

## 9. Some Active Endogenous Substances Involved in IPF Pathogeny

### 9.1. Fatty Acids

There are complex changes in fatty acid metabolism in pulmonary fibrosis [155]. In patients with IPF, an increase of about 53% in the total concentration of fatty acids in the serum was observed. There are conflicting data regarding the concentration of saturated fatty acids in BALF. Some authors found higher and others lower concentrations compared to the concentrations in healthy people of the same age [156,157]. The role of these lipids in the development of pulmonary fibrosis cannot be neglected. In experimental studies in mice, a diet rich in palmitic acid was associated with the development of pulmonary fibrosis [156]. This acid also increased apoptosis. Pulmonary fibrosis produced with bleomycin is exacerbated by the administration for three weeks of a diet rich in palmitic and stearic acid. A diet with a reduced amount of magnesium administered for 14 weeks in rats significantly increased the concentration of long-chain polyunsaturates such as 22:4n6 and 22:6n3 at the vascular and serum levels [158]. In the liver, zinc deficiency produced an accumulation of lipids. In this situation, the ratio between the amounts of different lipid substances also changes. The amount of alpha-linolenic acid and eicosapentaenoic acid increases significantly and the concentration of stearic and arachidonic acids decreases [159]. Supplementing the diet of patients with respiratory distress syndrome with omega-3 fatty acid had a beneficial effect on lung function [160].

### 9.2. Eicosanoids

Eicosanoids are a large group of active endogenous substances derived from unsaturated fatty acids. There are several groups of eicosanoids (prostaglandins, thromboxanes, prostacyclin, leukotrienes, lipoxins, resolvins, epoxy derivatives and others). All these groups of eicosanoids have membrane receptors and are involved in numerous normal and pathological processes throughout the human and animal body, including the lungs. Eicosanoids are involved in the development of some forms of pulmonary fibrosis [161]. Balance between the different groups of eicosanoids is essential in this direction. Since the factors that stimulate or inhibit the production of different categories of eicosanoids are different, the involvement of these active endogenous lipids in the production of idiopathic pulmonary fibrosis and other forms of fibrosis is particularly complex and still insufficiently understood. There are two groups of leukotrienes, leukotriene B4 and peptidoleukotrienes (LTC4, LTD4, LTE4), with all of these leukotrienes being synthesized from arachidonic acid. LTB4 is an important factor in the production of lung inflammation and in the migration of neutrophils [162]. It has been shown that the inhibition of leukotriene synthesis by leukotriene A4 hydrolase (LTA4H). The final step in LTB4 synthesis is mediated by leukotriene A4 hydrolase. This is a zinc bifunctional metalloenzyme with both hydrolase and aminopeptidase activity.

LTA4 hydrolase is a metalloenzyme that contains 1 mmol zinc/1 mmol of the enzyme. It was shown that an increase in this ratio in favor of zinc inhibits the activity of this hydrolase and implicitly the synthesis of LTA4 [163]. Besides the inhibition of LTA4 H, the addition of zinc in micromolar concentrations inhibited the activity of 15-lipoxygenase, the essential enzyme in the synthesis of lipoxins, in human polymorphonuclear leukocytes. Peptidoleukotrienes are involved in the pathogenesis of diabetic fibrosis. The administration of montelukast, a competitive antagonist at the CysLT1 leukotriene receptor level, during the induction of streptozotocin-induced diabetes mellitus in rats, significantly reduced the development of pulmonary fibrosis, pulmonary edema and leukocyte infiltration [164].

The synthesis of peptidoleukotrienes is inhibited only by high concentrations of zinc (over 1 mmol), which are difficult to reach in the human body. A higher concentration of zinc modifies the structure of membrane phospholipids, affecting the desaturation and elongation of the essential fatty acids, including those acids that are precursors of eicosanoids. Changing the ratio between the concentrations of zinc and copper also influences the unsaturated fatty acid composition of the phospholipids of cell membranes [165]. The association of zinc with CysLT1 receptor antagonists of peptidoleukotrienes (such as montelukast) increased the effect of these antagonists in experimental models of thermoalgesia and other pathological situations [166]. Influences of prostagandins on the development of pulmonary fibrosis are different. PGF2alpha is important in the development of pulmonary fibrosis. Suppression of the receptors for this prostaglandin leads to a reduction in the development of bleomycin-induced pulmonary fibrosis. On the contrary, 15-deoxy-Delta12,14-prostaglandin J2 (15d-PGJ2) (30 microg/kg i.p.) administration in bleomycin-induced pulmonary fibrosis in mice reduced lung neutrophil infiltration, fibrosis and mortality rate of animals [167]. Suppressing the activity of phospholipase A2 (PLA2) (the enzyme that releases from membrane phospholipids fatty acid precursors of eicosanoids) also decreases the development of experimental pulmonary fibrosis [168]. Other groups of eicosanoids, such as lipoxins, protectins, and resolvins, are considered pro-resolving factors. These agents reduce neutrophil infiltration, decrease inflammation, and diminish (or possibly prevent) the development of fibrosis in some pathological situations [169,170]. Specialized pro-resolving mediators are different groups of eicosanoids derived from omega-3 and omega-6 polyunsaturated fatty acids. The first known group of these eicosanoids was the group of lipoxins synthesized by the 15-lipoxygenase pathway (15-LOX) from arahidonic acid. Then other groups of eicosanoids involved in the resolution of acute inflammation and the control of the inflammatory process were identified. Resolvins, protectins and maresins have other groups of eicosanoids involved in the resolution of inflammation with an anti-inflammatory role and reduction of fibrosis, but they are derived from docosahexaenoic acid (DHA) and eicosapenatenoic acid (EPA) [171].

MgIG inhibits PLA2 and suppresses the stimulation of this enzyme by lipopolysaccharide (LPS). In this way, the release of arachidonic acid from membrane phospholipids decreases.

Besides this, some of the essential enzymes in the synthesis of some groups of eicosanoids are also inhibited. Existing data show that this magnesium compound inhibits Cyclooxygenase 1 (COX1) and Cyclooxygenase 2 (COX2) (the main enzymes in the synthesis of pro-inflammatory prostaglandins (both E and F) and also 5-LOX (which determines the synthesis of leukotrienes)) [70,172]. In acute lung injury in endotoxemia rats, administration of MgSO4 reduced the inflammatory response, PLA2 activity and eicosanoid synthesis [39]. Mg^2+^ also dose-dependently inhibited 5-LOX and the synthesis of leukotrienes in human polymorphonuclear leukocytes [173]. Zinc inhibited the stimulation of PLA2 and the release of arachidonic acid [174]. Unfortunately, there are no studies on the actions of this cation on PLA2 in the lungs, but since there are no significant differences between the phospholipases in the cell membranes of different organs, we consider that the action is the same. Zinc ions also inhibit PLA2 activity in vitro [174]. MAPK is involved in the activation of cytosolic PLA2 [175]. There may also be indirect actions of bivalent cations on PLA2 through the modulation of MAPK activity. Some studies have shown that the concentration of pulmonary prostaglandins does not change significantly in copper-deficient animals compared to the control group [176]. In the cytosol of lung cells, CuSO_4_·5H_2_O inhibited both the activity of 5-lipooxygenase (5-LOX) and that of 15-LOX, i.e., the synthesis of leukotrienes and lipoxins. The significance of this fact for the evolution of pulmonary fibrosis remains to be established [177].

It has been experimentally proven that blocking the synthesis of LTB4 by inhibiting LTA4H significantly reduces the development of pulmonary inflammation and fibrosis caused by the administration of lipopolysaccharide (LPS) [178]. Blocking CysLT1 receptors for peptidoleukotrienes determined a reduction in the NF-κB protein expression and production of pro-inflammatory cytokines [179]. Peptidoleukotriene antagonists such as montelukast reduce the development of fibrosis and are widely used in the therapy of bronchial asthma, some forms of bronchitis, and other diseases [164,180,181]. Bivalent cations have different influences compared to the action of CysLT1 antagonists in different pathological conditions. Thus, montelukast not only reduces the development of diabetic pulmonary fibrosis and bronchoconstriction, but also increases the threshold of thermoalgesic pain [166]. CuSO_4_ and other copper compounds inhibit the lipoxygenages that catalyze the hydroperoxidation of polyunsaturated fatty acids; the essential stage synthesizes more eicosanoid groups such as leukotrienes, lipoxins, hepoxilins and others [177]. AECII captures zinc through a process that is stimulated by arachidonic acid, which is the main precursor in the synthesis of eicosanoids [182].

### 9.3. Protein p53

The p53 protein is a suppressor of mitogenesis and a protein that has a role in reducing tumor development. Activation of the p53-dependent signaling pathway is involved in the senescence of alveolar cells and the development of pulmonary fibrosis [183]. P53-related signaling pathway activity increases with age in experimental animals [184]. Because p53 is a tumor suppressor gene, mutations in this gene are associated by some authors with increased incidence of lung cancers in patients with IPF [185]. This protein also activates autophagy in neoplastic cells. In alveolar epithelial cells of patients with IPF, the level of p53 is higher than in normal people of the same age [186]. In experimental studies, magnesium acetyltaurate has anti-apoptotic activity by reducing p53 activation by different factors [187]. On the other hand, magnesium superoxide dismutase expression in lungs is regulated by p53 [188]. In clinical studies, administration of magnesium (300 mg/day magnesium sulfate) for 3 months leads to downregulation of the p53 gene [189]. The p53 protein is a zinc-containing transcription factor.

The relationship between copper and zinc regarding this protein is complex. A low level of zinc amplifies the intracellular signaling of p53 [190]. Cu^2+^ displaces Zn^2+^ from the p53 protein [191]. An imbalance between the concentrations of zinc and copper could contribute to the progression of pulmonary fibrosis and to the development of lung cancer.

### 9.4. Sirtuin 1 (SIRT 1) and Sirtuin 3 (SIRT3)

Sirtuin 1 and the other sirtuins are a group of enzymes involved in the removal that remove the acyl from lysine residues. They belong to the group of NAD(+)-dependent protein deacetylases [192].

SIRT 1 is very important in cell proliferation. The activation of SIRT 1 at the lung level has an anti-senescent effect. Reducing the activity of this factor produces stimulation of cellular autophagy of alveolar epithelial type 2 cells and promotes pulmonary fibrosis [193]. The administration of magnesium lithospermate B (25–50 mg/kg/day) in experimental studies in rats increased the expression of SIRT1 [194]. Zinc activates the sirtuin signaling pathway [182,195]. In the alveolar cells of patients with IPF, the concentration of zinc is lower compared to the alveolar cells of normal people of the same age [151]. NF-kB is an essential element in the pathogenic mechanism of inflammation.

SIRT1 activation inhibits NF-kB activity and reduces the inflammatory process. By activating SIRT 1, zinc reduces NF-kB activity and implicitly the inflammatory process. Nrf2 is a translational factor with anti-inflammatory action whose activity is stimulated by SIRT 1 [196]. By increasing the activity and expression of SIRT 1, magnesium and zinc have anti-inflammatory action in pulmonary fibrosis. Deficiency in the zinc transporter SLC39A8 (ZIP8) of alveolar epithelial cells is involved in the increase in fibrogenesis in patients with IPF [197]. The data regarding the influence of copper on SIRT1 activity are not yet clear.

SIRT 3 is also important in the normal function of the AECs. This sirtuin detoxifies mitochondrial reactive oxygen species. Factors that decrease SIRT3 activity such as bleomycin, asbestosis, and others cause the appearance of pulmonary fibrosis [198].

The main implications of magnesium in the pathogenesis of pulmonary fibrosis are summarized in Table 1.

## 10. Apoptosis

The role of apoptosis changes in idiopathic pulmonary fibrosis is complex. On the one hand, increased apoptosis of alveolar cells is involved in the development of pulmonary fibrosis [201,202]; on the other hand, an increase in the apoptosis of pulmonary fibroblasts could slow down the development of pulmonary fibrosis. Bax and caspase 3 are essential proapoptotic proteins. Caspase 3 is a very important protein for apoptosis throughout the body, including the lung. Alveolar epithelial cell destruction as a result of the action of various harmful factors is an important element in the development of idiopathic pulmonary fibrosis. Pirfenidone inhibits caspase 3. Through this mechanism, this drug reduces apoptosis induced by TNF-α [203]. Nintedanib has a potent selective removal effect of senescent cells. This effect was observed in the case of bleomycin-induced senescence of human pulmonary fibroblasts [204]. The action of bivalent cations at the level of proapoptotic and antiapoptotic proteins still requires studies to be clarified.

Expression of B-cell lymphoma 2 (Bcl-2) protein was increased by Magnesium-L-threonate after 3 months’ administration in mice. At the same time, magnesium upregulated Bcl-2. The experimental administration of magnesium reduced the Bax level (which increases in hypoxia conditions) and normalized the Bax/Bcl ratio [199]. MgIG also inhibited apoptosis increased by some toxicants [200]. There is no data to show that the inhibition of apoptosis and autophagy occurs through the same mechanism. This compound (50 mg/kg/day) decreased also the expression levels of caspase-3, not only Bax levels. Copper increases NF-kB activity [66].

Increasing intracellular copper concentration induces apoptosis and enhances autophagy [205,206]. This biometal downregulated Bcl2 and upregulated proapoptotic proteins Bax and caspase 3 [207]. Activation of the caspase pathway is an important mechanism by which copper induces apoptosis [208]. In experimental models of pulmonary fibrosis produced with bleomycin, the administration of tetrathiomolybdate, a substance that lowers copper concentration, produced a reduction in the development of pulmonary fibrosis [209].

In patients with IPF, AEC apoptosis is significantly higher than in normal people. The expression of caspase-3 and Bax, both proapoptotic proteins, is increased in these patients [210]. Some drugs such as pirfenidone do not significantly change the concentration of caspase 3 in serum and bronchoalveolar lavage fluid in patients with IPF [211].

On the other hand, fibroblast apoptosis is a crucial process for reducing the progression of pulmonary fibrosis. The resistance of fibroblasts from patients with IPF to apoptosis is higher than that of normal fibroblasts.

The main implications of zinc and copper in the pathogenesis of pulmonary fibrosis are summarized in Table 2.

## 11. Genetics

A number of genes are involved in the development of IPF. An example is the fact that the polymorphism of the TGF-beta1 gene is associated with the progression of IPF [212]. A direct action of zinc, magnesium or copper at the level of the genes involved in development of IPF is not known. Some authors state that IPF is a telomere-mediated disease. Telomerase gene mutations are associated with IPF [213]

Without a doubt, the interaction between genetic and epigenetic factors is also important in the pathogenesis of this disease (as in the case of other diseases in the human clinic), but there are no data regarding the mechanism by which these bivalent cations can act at the level of the genes potentially involved in the development of IPF.

## 12. Therapeutics

For the time being, there are few data on the therapeutic use of magnesium and zinc in the therapy of pulmonary fibrosis, and the existing experimental data must be carefully translated into clinical practice.

In the case of IPF, several drugs such as Prednisone, Azathioprine, and N-Acetylcysteine (in monotherapy or in therapeutic combinations) have been recommended but have not been satisfactory [214]. Current clinical guidelines (ATS) recommend the use of pirfenidone and nintedanib (a Tyrosine Kinase Inhibitor) in the treatment of IPF [215]

Pirfenidone reduces the development of experimental lung fibrosis induced by bleomicine by increasing expression of Nrf2 and glutation peroxidase and decreasing the level of Il-6 in serum and BALF. The MDA and ROS concentration is also reduced [216]. Zinc decreases the synthesis of Il-6 and other pro-inflammatory interleukins, decreases the expression of TGF-β1 and the formation of MDA, and increases the expression of Nrf2. Tetrandrine-loaded zinc–alginate nanogels reduced experimental fibrosis in rats. The authors believe that the product offers hope for clinical use, but it is not clear what the contribution of tetradrine or zinc is to this effect [217]. Part of the therapeutic effect of N-acetylcysteine and pirfenidone is produced by reducing oxidative stress [218,219]. Zinc also has a similar effect. We believe that in patients with pulmonary fibrosis, the combination of zinc in therapy would be beneficial, increasing the effectiveness of drugs that reduce the development of fibrosis. This is a hypothesis that needs to be verified through clinical trials and, as yet, has not been verified in therapeutic practice. However, the existing data strongly support it.

The doses of magnesium and zinc associated with the administration of pirfeniperformed must be 400 mg/day of magnesium and 15 mg/day of zinc for an average-weight adult. The administration of the two cations should be done throughout the therapy with pirfenidone. No adverse effects have been reported on the combination of pirfenidone or nintedanib with magnesium or zinc.

The periodic dosing of magnesium must aim not only at the correction of plasma deficits but also at the permanent maintenance of the intracellular concentration of this cation within normal limits. At these doses, no adverse effects of these two bivalent cations were reported. In the case of patients with chronic kidney disease, clinical reports have shown that magnesium administration is safe. In patients with renal failure, routine hemodialysis induces a decline in plasma magnesium concentration [220], and administration of magnesium to normalize serum concentrations of this cation is beneficial. Mg deficiency worsens kidney injury [221].

Magnesium lithospermate B (10 mg kg/L/day) administered for 4 weeks in an experimental fibrosis model in mice significantly reduced the development of fibrosis and inflammation. The authors consider that it is possible to use this magnesium compound in the human clinic [222]. In patients with cystic fibrosis, supplementing the diet with magnesium improved lung function [223].

In addition to their effect in pulmonary fibrosis, magnesium and zinc have a beneficial effect in pulmonary hypertension and in right ventricular failure. Pulmonary hypertension is a severe complication of pulmonary fibrosis. Mg^2+^ supplementation reduced pulmonary arterial pressure and right heart hypertrophy. Magnesium inhibits pulmonary arterial smooth muscle cell proliferation and migration [224]. Nebulized magnesium sulfate therapy is effective in reducing persistent pulmonary hypertension of the newborn [225]. In an experimental study, zinc chloride injection (5 mg/kg) attenuates monocrotaline-induced pulmonary hypertension in rats [226]. Magnesium lithospermate B (MLB) prevented the elevation in right ventricular systolic pressure in rats by downregulating NOX (NOX2 and NOX4) protein levels, and suppressing the phosphorylation of ERK [227]. Pulmonary fibrosis causes not only pulmonary hypertension but also right ventricular failure. A large clinical study showed that magnesium depletion is associated with congestive heart failure [228]. Low serum magnesium levels, high copper, and low zinc were associated with a higher risk of developing major adverse cardiovascular events including ventricular failure [229].

## 13. Conclusions

Although the knowledge of the involvement of these divalent cations in the pathogenesis of pulmonary fibrosis is still incomplete and existing experimental data should be translated with caution into clinical practice, there are strong arguments for the involvement of Mg, Zn and Cu in the pathogenic mechanism of pulmonary fibrosis.

We consider that the association of zinc and magnesium with the treatment of nintedanib and pirfenidone is important. The two drugs, the most used today in the therapy of pulmonary fibrosis, slow down the development of this pathological process without being able to stop its evolution [215]. We believe that the association of zinc and magnesium with the drugs used in the treatment of pulmonary fibrosis would increase the therapeutic efficiency of these.

The incidence of pulmonary cancer is much higher in healthy individuals with IPF than in the general population. The reasons why a much higher proportion of patients with IPF develop lung cancer compared to the general population are not well understood. A significantly lower level of plasma zinc is positively associated with increased risk of developing lung cancer [230], and a high copper/zinc ratio is associated with an increased risk of this type of cancer [32]. Some authors have suggested that the plasma copper level and the copper/zinc ratio could be used to predict the development of lung cancer [230].

We consider that a low level of zinc and a higher than normal copper/zinc ratio could indicate which of the IPF patients have an increased risk of developing lung cancer. A multi-center study demonstrated that among patients receiving anti-cancer therapy with immune checkpoint blockers, elevated serum magnesium levels (Mg^2+^ ≥ 0.79 mmol/L) exhibited significantly better clinical outcomes compared to other patients receiving the same therapy but with low serum magnesium levels [231].

The zinc and magnesium deficiency must be immediately corrected in all IPF patients. An increased intake of zinc was positively associated with a significant reduction in the occurrence of lung cancer [232]

Regarding the frequent association between IPF and lung cancer, we consider a hypothesis that normalizing the copper/zinc ratio and correcting the reduced zinc level in the early stages of IPF could reduce the incidence of lung cancer in these patients.

As future research directions, it is strictly necessary to check if the existing experimental data can be translated into clinical practice. More data are needed regarding the early stages of pulmonary fibrosis and the concentrations of bivalent cations, as well as the possibility that by correcting cationic imbalances in people with an increased incidence of pulmonary fibrosis, the incidence of this disease can be reduced. Such clinical research is needed to show whether or not the early correction of imbalances in the concentrations of bivalent cations determines a reduction in the speed of evolution of this very serious chronic disease. There are no studies showing the influence of divalent cations on the development of pulmonary fibrosis in people of different ages.

### Limitations

Translating experimental results into human clinical practice has two major difficulties: The first is given by the fact that experimental models of pulmonary fibrosis have a well-defined onset in time and a short duration of fibrosis production. In the case of human fibrosis, the evolution is much longer, and the beginning of the development of the fibrosis process is difficult to establish. Another problem is that experimental fibrosis is induced by a single factor (e.g., bleomycin), but human pulmonary fibrosis develops under the action of a complex of factors, and in the case of IPF, some are probably still unknown.

## Figures and Tables

**Table 1:** The implications of magnesium in the pathogenesis of pulmonary fibrosis.

	Mg^2+^ Actions
Oxidative stress	In animal studies, Mg^2+^ significantly reduced oxidative stress and lung lesions associated with it [39]. Mg^2+^ inhibits nuclear factor-kappaB (NF-κB) activity [40,41]
Inflammation	Magnesium isoglycyrrhizinate (MgIG) reduces the formation of pro-inflammatory factors determined by LPS [51]. MgIG significantly reduced the concentration of IL-6 and TNF-α at the lung and serum levels in rats [58]. Low magnesium concentration causes an increase in the expression of p38-mitogen-activated protein kinase (MAPK) [60] Magnesium citrate 300 mg/day in patients with COPD significantly reduced the level of CRP [73]. Magnesium in cell culture increased the activity of MAPK, ERK 1 and ERK 2 [69]. In a double-blinded, placebo-controlled trial, administration of 250 mg/day magnesium oxide for 6 weeks downregulated gene expression levels of interleukin-8 (c) and TNF-α [61].
Mitochondrial dysfunctions	Mg^2+^ is essential for the activity of ATP and the production of energy at the mitochondrial level.
Excessive extracellular matrix accumulation	Magnesium isoglycyrrhizinate inhibited the activity of MMP-9 in rats with lung injury [82]. In human cell cultures, magnesium concentrations (0–3.0 mmol/L) significantly reduced the production of MMP2 stimulated by homocysteine [83]
Fibroblasts and myofibroblasts differentiation and fibrosis	Mg-deficient diet in rats induced a19% increase in collagen synthesis and deposition [96]. MgIG reduced the pulmonary concentration of TGF-1 beta in radiation-induced experimental pulmonary fibrosis and decreased fibrosis progression [99]. Magnesium lithospermate B (50 mg/kg/day for 7 days), in animal studies, had an antifibrotic effect [100]. Magnesium inhibits the differentiation of pulmonary fibroblasts by influencing the MAPK/Akt/Nox4 biological signal transduction pathway [116].
Alveolar macrophage	The decrease in magnesium concentration in the culture medium of the alveolar macrophages determines the increase in the synthesis of nitric oxide through the stimulation of iNOS [130]
Alveolar epithelial cells	Magnesium hydride (MgH_2_) diminishes LPS induced inflammatory reaction, apoptosis, and barrier breakdown in alveolar epithelial cells [100] MgH_2_ also suppressed barrier breakdown by upregulating the expression of occludin [150].
Some active endogenous substances involved in IPF pathogeny	MgIG inhibits PLA2 and suppresses the stimulation of this enzyme by lipopolysaccharide (LPS). MgIG compound inhibits Cyclooxygenase 1 (COX1) and Cyclooxygenase 2 (COX2) (the main enzymes in the synthesis of pro-inflammatory eicosanoids) [70,172]. In clinical studies, administration of magnesium (300 mg/day magnesium sulfate) for 3 months led to downregulation of p53 gene [189]. Magnesium lithospermate B (25–50 mg/kg/day) in experimental studies in rats increased the expression of SIRT1 [194]
Apoptosis	Mg^2+^ reduced the Bax level (which increases in hypoxia conditions) and normalized the Bax/Bcl ratio [199]. MgIG also inhibited apoptosis increased by some toxicants [200].

**Table 2:** Zinc and copper implications in the pulmonary fibrosis pathogenesis.

	Zn^2+^ and Cu^2+^ Actions
Oxidative stress	Zinc deficiency exacerbates oxidative stress in the lungs, increases the production of ROS and decreases the reduction in the expression of Nrf2 [38]. Zinc increases the level of GSH [45]. Zinc aspartate in acute lung injury significantly reduces the pulmonary level of malondialdehyde (MDA), nitric oxide, and myeloperoxidase [46]. Zinc sulfate in the case of lung injury produced an increase in SOD levels and alleviated lung injury [49]. The copper(II) catalyzed GSH oxidation [51]. The increase in Cu concentration in the lungs is associated with increased oxidative stress in patients with cystic fibrosis [50].
Inflammation	Zinc has anti-inflammatory actions [55]. Zinc alleviates inflammation induced by lipopolysaccharide (LPS) and other pro-inflammatory factors [56]. The main way through which zinc regulates biological signal transduction mediated by ERK1/2 is the inhibition of phosphatases that determine the dephosphorylation of ERK1 and ERK2 [72]. In animal studies, copper in drinking water for 16 weeks showed an increase in oxidative stress and pro-inflammatory factors at the lung level, simultaneously with an intensification of copper accumulation in the lungs [75]. A copper/zinc ratio higher than normal has a pro-inflammatory action [76].
Mitochondrial dysfunctions	Copper concentrations higher than normal it causes mitochondrial dysfunction. Copper induces an increased mitochondrial deposition of lipids [79]. Copper produces swelling and decreases the mitochondrial membrane potential [80].
Excessive extracellular matrix accumulation	In human clinic, serum zinc levels are negatively correlated with the concentration of MMP-2, MMP-9 [84]. In animal studies, the addition of zinc to the normal diet or to the high-fat diet significantly reduced MMP2 and MMP9’s expressions [85]. Copper-methionine administration in cold-stressed broilers decreased the concentration of MMP-2 [87].
Fibroblasts and myofibroblasts differentiation and fibrosis	Zinc in drinking water reduced collagen accumulation in the lungs of animals experimentally intoxicated with CCl_4_ by the inhibition of the lung prolyl hydroxylase activity [101]. In animal studies, zinc reduced the deposition of collagen in the lungs [102]. A copper-dependent monoamine oxidase is also involved in the development of fibrosis in various organs [104]. The main mechanism of action of intracellular copper ions is the activation of LOXL(lysyl oxidase-like) [105]. LOXL is an essential enzyme in collagen synthesis and the formation of the extracellular matrix. Amine oxidase copper-containing-3 (AOC3) is an enzyme with a role in the development of pulmonary fibrosis [110]. Exposure to CuSO4 (100 μM concentration) in vitro produces a decrease in the viability of both alveolar cells and bronchoepithelial cells [113]. Copper also increases the autophagy [79] and inhibits the acetylcholinesterase [115].
Alveolar macrophage	Zinc deficiency increased the macrophage infiltration [56]. Exposure of alveolar macrophages to copper oxide increases the generation of ROS in these cells [97]. The copper transporter ATP7A was identified in macrophages [134]. Exposure of animals to CuSO_4_ in air produces an increase in the plasma concentration of isoprostane F2 and an increase in the total number of neutrophils and macrophages in BALF [135].
Alveolar epithelial cells	Deficiency of a zinc transporter, SLC39A8 (ZIP8), has been identified in AEC2s in patients with IPF [151]. In AECs, copper oxide increases oxidative stress and produces morphological changes. Copper oxide induces mitochondrial damage [152].
Some active endogenous substances involved in IPF pathogeny	Zinc in micromolar concentrations inhibited the activity of 15-lipoxygenase, the essential enzyme in the synthesis of lipoxins, in human polymorphonuclear leukocytes [163]. Ratio between the concentrations of zinc and copper also influences the unsaturated fatty acid composition of the phospholipids of cell membranes [165]. Zinc inhibited the stimulation of PLA2 and the release of arachidonic acid [174]. AECII captures zinc through a process that is stimulated by arachidonic acid [182] CuSO_4_·5H_2_O inhibited both the activity of 5-lipooxygenase (5-LOX) and that of 15-LOX, i.e., the synthesis of leukotrienes and lipoxins [177]. CuSO_4_ and other copper compounds inhibit the lipoxygenases that catalyze the hydroperoxidation of polyunsaturated fatty acids [177]. Cu^2+^ displaces Zn^2+^ from p53 protein [191]. Zinc activates the sirtuin signaling pathway [195]. By activating SIRT 1, zinc reduces NF-kB activity [196].
Apoptosis	Increasing intracellular copper concentration induces apoptosis and enhances autophagy [205,206]. Copper activates the caspase pathway. It is an important mechanism by which copper induces apoptosis [208].

## Data Availability

No new data were created or analyzed in this study.

## References

[1] Richeldi L., Collard H.R., Jones M.G. (2017). Idiopathic pulmonary fibrosis. Lancet.

[2] Niccoli T., Partridge L. (2012). Ageing as a Risk Factor for Disease. Curr. Biol..

[3] Giriyappagoudar M., Vastrad B., Horakeri R., Vastrad C. (2023). Study on Potential Differentially Expressed Genes in Idiopathic Pulmonary Fibrosis by Bioinformatics and Next-Generation Sequencing Data Analysis. Biomedicines.

[4] Martinez F.J., Collard H.R., Pardo A., Raghu G., Richeldi L., Selman M., Swigris J.J., Taniguchi H., Athol U., Wells A.U. (2017). Idiopathic pulmonary fibrosis. Nat. Rev. Dis. Primers.

[5] Raghu G., Collard H.R., Egan J.J., Martinez F.J., Behr J., Brown K.K., Colby T.V., Cordier J.-F., Flaherty K.R., Lasky J.A. (2011). An official ATS/ERS/JRS/ALAT statement: Idiopathic pulmonary fibrosis: Evidence-based guidelines for diagnosis and management. Am. J. Respir. Crit. Care Med..

[6] Chanda D., Otoupalova E., Smith S.R., Volckaert T., De Langhe S.P., Thannickal V.J. (2019). Developmental pathways in the pathogenesis of lung fibrosis. Mol. Asp. Med..

[7] Raghu G., Chen S.Y., Yeh W.S., Maroni B., Li Q., Lee Y.C. (2014). Idiopathic pulmonary fibrosis in US Medicare beneficiaries aged 65 years and older: Incidence, prevalence, and survival, 2001–11. Lancet Respir. Med..

[8] American Thoracic Society (2000). Idiopathic pulmonary fibrosis: Diagnosis and treatment. International consensus statement. American Thoracic Society (ATS), and the European Respiratory Society. Am. J. Respir. Crit. Care Med..

[9] Chianese M., Screm G., Salton F., Confalonieri P., Trotta L., Barbieri M., Ruggero L., Mari M., Reccardini N., Geri P. (2024). Pirfenidone and Nintedanib in Pulmonary Fibrosis: Lights and Shadows. Pharmaceuticals.

[10] Finnerty J.P., Ponnuswamy A., Dutta P., Abdelaziz A., Kamil H. (2021). Efficacy of antifibrotic drugs, nintedanib and pirfenidone, in treatment of progressive pulmonary fibrosis in both idiopathic pulmonary fibrosis (IPF) and non-IPF: A systematic review and meta-analysis. BMC Pulm. Med..

[11] Wang D., Ma Y., Tong X., Zhang Y., Fan H. (2020). Diabetes Mellitus Contributes to Idiopathic Pulmonary Fibrosis: A Review From Clinical Appearance to Possible Pathogenesis. Front. Public Health.

[12] Mzimela N., Dimba N., Sosibo A., Khathi A. (2024). Evaluating the impact of type 2 diabetes mellitus on pulmonary vascular function and the development of pulmonary fibrosis. Front. Endocrinol..

[13] Lawson W.E., Crossno P.F., Polosukhin V.V., Roldan J., Cheng D.-S., Lane K.B., Blackwell T.R., Xu C., Markin C., Ware L.B. (2008). Endoplasmic reticulum stress in alveolar epithelial cells is prominent in IPF: Association with altered surfactant protein processing and herpesvirus infection. Am. J. Physiol. Lung Cell Mol. Physiol..

[14] Molyneaux P.L., Cox M.J., Willis-Owen S.A.G., Mallia P., Russell K.E., Russell A.-M., Murphy E., Johnston S.L., Schwartz D.A., Wells A.U. (2014). The role of bacteria in the pathogenesis and progression of idiopathic pulmonary fibrosis. Am. J. Respir. Crit. Care Med..

[15] Goto T. (2018). Measuring Surgery Outcomes of Lung Cancer Patients with Concomitant Pulmonary Fibrosis: A Review of the Literature. Cancers.

[16] Kinoshita T., Goto T. (2019). Molecular Mechanisms of Pulmonary Fibrogenesis and Its Progression to Lung Cancer: A Review. Int. J. Mol. Sci..

[17] Romani A.M.P., Vink R., Nechifor M. (2011). Intracellular magnesium homeostasis. Magnesium in the Central Nervous System.

[18] Micke O., Vormann J., Kraus A., Kisters K. (2021). Serum magnesium: Time for a standardized and evidence-based reference range. Magnes. Res..

[19] Rosanoff A., West C., Elin R.J., Micke O., Baniasadi S., Barbagallo M., Campbell E., Cheng F.-C., Costello R.B., Gamboa-Gomez C. (2022). Recommendation on an updated standardization of serum magnesium reference ranges. Eur. J. Nutr..

[20] Barbagallo M., Dominguez L.J. (2010). Magnesium and aging. Curr. Pharm. Des..

[21] Thannickal V.J. (2013). Mechanistic Links between Aging and Lung Fibrosis. Biogerontology.

[22] Lutsenko S. (2010). Human copper homeostasis: A network of interconnected pathways. Curr. Opin. Nephrol. Hypertens..

[23] Lech T., Sadlik J.K. (2007). Copper concentration in body tissues and fluids in normal subjects of southern Poland. Biol. Trace Elem. Res..

[24] Lin J., Xu F., Zhang C., Wu B. (2022). The effect of some trace elements on the expression of telomerase gene in lung cancer. Cell. Mol. Biol..

[25] Hasanato R.M.W. (2020). Trace elements in type 2 diabetes mellitus and their association with glycemic control. Afr. Health Sci..

[26] Bost M., Houdart S., Oberli M., Kalonji E., Huneau J.F., Margaritis I. (2016). Dietary copper and human health: Current evidence and unresolved issues. J. Trace Elem. Med. Biol..

[27] Prasad A.P. (2013). Discovery of human zinc deficiency: Its impact on human health and disease. Adv. Nutr..

[28] Warthon-Medina M., Moran V.H., Stammers A.-L., Dillon S., Qualter P., Nissensohn M., Serra-Majem L., Lowe N.M. (2015). Zinc intake, status and indices of cognitive function in adults and children: A systematic review and meta-analysis. Eur. J. Clin. Nutr..

[29] Khan N.A., Singla M., Samal S., Lodha R., Medigeshi G.R. (2020). Respiratory Syncytial Virus-Induced Oxidative Stress Leads to an Increase in Labile Zinc Pools in Lung Epithelial Cells. mSphere.

[30] Bargagli E., Monaci F., Bianchi N., Bucci C., Rottoli P. (2008). Analysis of trace elements in bronchoalveolar lavage of patients with diffuse lung diseases. Biol. Trace Elem. Res..

[31] Wang Y., Sun Z., Li A., Zhang Y. (2019). Association between serum zinc levels and lung cancer: A meta-analysis of observational studies. World J. Surg. Oncol..

[32] Zhang L., Shao J., Tan S.W., Ye H.P., Shan X.Y. (2022). Association between serum copper/zinc ratio and lung cancer: A systematic review with meta-analysis. J. Trace Elem. Med. Biol..

[33] Moss B.J., Ryter S.W., Rosas I.O. (2022). Pathogenic Mechanisms Underlying Idiopathic Pulmonary Fibrosis. Annu. Rev. Pathol..

[34] Wynn T.A. (2011). Integrating mechanisms of pulmonary fibrosis. J. Exp. Med..

[35] Matsuzawa Y., Kawashima T., Kuwabara R., Hayakawa S., Irie T., Yoshida T., Rikitake H., Wakabayashi T., Okada N., Kawashima K. (2015). Change in serum marker of oxidative stress in the progression of idiopathic pulmonary fibrosis. Pulm. Pharmacol. Ther..

[36] Yamazaki C., Hoshino J., Hori Y., Sekiguchi T., Miyauchi S., Mizuno S., Hori K. (1997). Effect of lecithinized-superoxide dismutase on the interstitial pneumonia model induced by bleomycin in mice. Jpn. J. Pharmacol..

[37] Kurys E., Kurys P., Kuźniar A.R., Kieszko R. (2001). Analysis of antioxidant enzyme activity and magnesium level in chronic obstructive pulmonary disease (COPD). Ann. Univ. Marie Curie Sklodowska Med..

[38] Luan R., Luo M., Ding D., Su X., Yang J. (2024). Zinc deficiency increases lung inflammation and fibrosis in obese mice by promoting oxidative stress. Biochim. Biophys. Acta Gen. Subj..

[39] Lee C.Y., Jan W.C., Tsai P.S., Huang C.J. (2011). Magnesium sulfate mitigates acute lung injury in endotoxemia rats. J. Trauma.

[40] Wang J., Ma C., Wang S. (2015). Effects of acteoside on lipopolysaccharide-induced inflammation in acute lung injury via regulation of NF-κB pathway in vivo and in vitro. Toxicol. Appl. Pharmacol..

[41] Jiang J., Chen Q., Chen X., Li J., Li S., Yang B. (2020). Magnesium sulfate ameliorates sepsis-induced diaphragm dysfunction in rats via inhibiting HMGB1/TLR4/NF-κB pathway. NeuroReport.

[42] Zhang L., Yang L., Xie X., Zheng H., Zheng H., Zhang L., Liu C., Piao J.-G., Li F. (2021). Baicalin Magnesium Salt Attenuates Lipopolysaccharide-Induced Acute Lung Injury via Inhibiting of TLR4/NF-*κ* B Signaling Pathway. J. Immunol. Res..

[43] Zhang Z., Weichenthal S., Kwong J.C., Burnett R.T., Hatzopoulou M., Jerrett M., van Donkelaar A., Bai L., Martin R.V., Copes R. (2021). A Population-Based Cohort Study of Respiratory Disease and Long-Term Exposure to Iron and Copper in Fine Particulate Air Pollution and Their Combined Impact on Reactive Oxygen Species Generation in Human Lungs. Environ. Sci. Technol..

[44] Lu S.C. (2013). Glutathione synthesis. Biochim. Biophys. Acta.

[45] Walther U.I., Walther S.C., Temrück O. (2007). Effect of enlarged glutathione on zinc-mediated toxicity in lung-derived cell lines. Toxicol. Vitr..

[46] Türüt H., Kurutas E.B., Bulbuloglu E., Yasim A., Ozkaya M., Onder A., Imrek S.S. (2009). Zinc aspartate alleviates lung injury induced by intestinal ischemia-reperfusion in rats. J. Surg. Res..

[47] Sacan O., Turkyilmaz I.B., Bayrak B.B., Mutlu O., Akev N., Yanardag R. (2016). Zinc supplementation ameliorates glycoprotein components and oxidative stress changes in the lung of streptozotocin diabetic rats. Biometals.

[48] Gursel F.E., Tekeli S.K. (2009). The effects of feeding with different levels of zinc and chromium on plasma thiobarbituric acid reactive substances and antioxidant enzymes in rats. Pol. J. Vet. Sci..

[49] Jing Y.L., Zhao C.X., Duan G.X., Wang Y.L., Hu Y.Q., Zhang L.Y. (2006). Therapeutic effect of zinc sulfate on lung injury during superior mesenteric artery occlusion(SMAO) shock. Zhongguo Ying Yong Sheng Li Xue Za Zhi.

[50] Kouadri A., Cormenier J., Gemy K., Macari L., Charbonnier P., Richaud P., Michaud-Soret I., Alfaidy N., Benharouga M. (2021). Copper-Associated Oxidative Stress Contributes to Cellular Inflammatory Responses in Cystic Fibrosis. Biomedicines.

[51] Ngamchuea K., Batchelor-McAuley C., Richard G., Compton R.G. (2016). The Copper(II)-Catalyzed Oxidation of Glutathione. Chemistry.

[52] Koudstaal T., Wijsenbeek M.S. (2023). Idiopathic pulmonary fibrosis. Presse Med..

[53] May R.D., Fung M. (2015). Strategies targeting the IL-4/IL-13 axes in disease. Cytokine.

[54] Peng L., Wen L., Shi Q.-F., Gao F., Huang B., Meng J., Hu C.-P., Wang C.-M. (2020). Scutellarin ameliorates pulmonary fibrosis through inhibiting NF-κB/NLRP3-mediated epithelial-mesenchymal transition and inflammation. Cell Death Dis..

[55] Prasad A.S. (2007). Zinc: Mechanisms of host defense. J. Nutr..

[56] Liu M.-J., Bao S., Gálvez-Peralta M., Pyle C.J., Rudawsky A.C., Pavlovicz R.E., Killilea D.W., Li C., Nebert D.W., Wewers M.D. (2013). ZIP8 regulates host defense through zinc-mediated inhibition of NF-κB. Cell Rep..

[57] Biaggio V.S., Pérez Chaca M.V., Valdéz S.R., Gómez N.N., Gimenez M.S. (2010). Alteration in the expression of inflammatory parameters as a result of oxidative stress produced by moderate zinc deficiency in rat lung. Exp. Lung Res..

[58] Yang Y., Huang L., Tian C., Qian B. (2021). Magnesium isoglycyrrhizinate inhibits airway inflammation in rats with chronic obstructive pulmonary disease. BMC Pulm. Med..

[59] Chinju A., Moriyama M., Kakizoe-Ishiguro N., Chen H., Miyahara Y., Rafiul Haque A.S.M., Furusho K., Sakamoto M., Kai K., Kibe K. (2022). CD163+ M2 Macrophages Promote Fibrosis in IgG4-Related Disease Via Toll-like Receptor 7/Interleukin-1 Receptor-Associated Kinase 4/NF-κB Signaling. Arthritis Rheumatol..

[60] Onuma S., Manabe A., Yoshino Y., Matsunaga T., Asai T., Ikari A. (2021). Upregulation of Chemoresistance by Mg^2+^ Deficiency through Elevation of ATP Binding Cassette Subfamily B Member 1 Expression in Human Lung Adenocarcinoma A549 Cells. Cells.

[61] Ahmadi S., Naderifar M., Samimi M., Mirhosseini N., Amirani E., Aghadavod E., Asemi Z. (2018). The effects of magnesium supplementation on gene expression related to inflammatory markers, vascular endothelial growth factor, and pregnancy outcomes in patients with gestational diabetes. Magnes. Res..

[62] Sonaglioni A., Caminati A., Lipsi R., Lombardo M., Harari S. (2021). Association between C-reactive protein and carotid plaque in mild-to-moderate idiopathic pulmonary fibrosis. Intern. Emerg. Med..

[63] Cottin V., Valenzuela C. (2024). C-reactive protein as a candidate biomarker in fibrotic interstitial lung disease. Respirology.

[64] Zanforlini B.M., Ceolin C., Trevisan C., Alessi A., Seccia D.M., Noale M., Maggi S., Guarnieri G., Vianello A., Sergi G. (2022). Clinical trial on the effects of oral magnesium supplementation in stable-phase COPD patients. Aging Clin. Exp. Res..

[65] Lominadze D., Saari J.T., Percival S.S., Schuschke D.A. (2004). Proinflammatory effects of copper deficiency on neutrophils and lung endothelial cells. Immunol. Cell Biol..

[66] Persichini T., Percario Z., Mazzon E., Colasanti M., Cuzzocrea S., Musci G. (2006). Copper activates the NF-κB pathway in vivo. Antioxid. Redox Signal.

[67] Kennedy T., Ghio A.J., Reed W., Samet J., Zagorski J., Quay J., Carter J., Dailey L., Hoidal J.R., Devlin R.B. (1998). Copper-dependent inflammation and nuclear factor-κB activation by particulate air pollution. Am. J. Respir. Cell Mol. Biol..

[68] Touyz R.M., Yao G. (2003). Modulation of vascular smooth muscle cell growth by magnesium-role of mitogen-activated protein kinases. J. Cell. Physiol..

[69] Salucci S., Giordani M., Betti M., Valentini L., Gobbi P., Mattioli M. (2024). The in vitro cytotoxic effects of natural (fibrous epsomite crystals) and synthetic (Epsom salt) magnesium sulfate. Microsc. Res. Tech..

[70] Xie C., Li X., Zhu J., Wu J., Geng S., Zhong C. (2019). Magnesium isoglycyrrhizinate suppresses LPS-induced inflammation and oxidative stress through inhibiting NF-κB and MAPK pathways in RAW264.7 cells. Bioorg Med. Chem..

[71] Nuttall J.R., Oteiza P.I. (2012). Zinc and the ERK kinases in the developing brain. Neurotox. Res..

[72] Azriel-Tamir H., Sharir H., Schwartz B., Hershfinkel M. (2004). Extracellular zinc triggers ERK-dependent activation of Na+/H+ exchange in colonocytes mediated by the zinc-sensing receptor. J. Biol. Chem..

[73] Liu Y., Xiao Y., Liu J., Feng L., Kang Y.J. (2018). Copper-induced reduction in myocardial fibrosis is associated with increased matrix metalloproteins in a rat model of cardiac hypertrophy. Metallomics.

[74] Boilan E., Winant V., Dumortier E., Piret J.-P., Bonfitto F., Osiewacz H.D., Debacq-Chainiaux F., Toussaint O. (2013). Role of p38MAPK and oxidative stress in copper-induced senescence. Age.

[75] Gaun S., Ali S.A., Singh P., Patwa J., Flora S.J.S., Datusalia A.K. (2023). Melatonin ameliorates chronic copper-induced lung injury. Environ. Sci. Pollut. Res. Int..

[76] Janssen R., de Brouwer B., Jan H., Wouters E.F. (2018). Copper as the most likely pathogenic divergence factor between lung fibrosis and emphysema. Med. Hypotheses.

[77] Bueno M., Calyeca J., Rojas M., Mora A.M. (2020). Mitochondria dysfunction and metabolic reprogramming as drivers of idiopathic pulmonary fibrosis. Redox Biol..

[78] Kim S.J., Cheresh P., Jablonski R.P., Morales-Nebreda L., Cheng Y., Hogan E. (2016). Mitochondrial catalase overexpressed transgenic mice are protected against lung fibrosis in part via preventing alveolar epithelial cell mitochondrial DNA damage. Free Radic. Biol. Med..

[79] Zhong C.C., Zhao T., Hogstrand C., Chen F., Song C.C., Luo Z. (2022). Copper (Cu) induced changes of lipid metabolism through oxidative stress-mediated autophagy and Nrf2/PPARγ pathways. J. Nutr. Biochem..

[80] Mashayekhi V.K., Hashemzaei M., Tabrizian K., Shahraki J., Hosseini M.J. (2015). Mechanistic approach for the toxic effects of perfluorooctanoic acid on isolated rat liver and brain mitochondria. Hum. Exp. Toxicol..

[81] McKeown S., Richter A.G., O’Kane C., McAuley D.F., Thickett D.R. (2009). MMP expression and abnormal lung permeability are important determinants of outcome in IPF. Eur. Respir. J..

[82] Xiao Z.W., Zhang W., Ma L., Qiu Z.W. (2014). Therapeutic effect of magnesium isoglycyrrhizinate in rats on lung injury induced by paraquat poisoning. Eur. Rev. Med. Pharmacol. Sci..

[83] Guo H., Lee J.-D., Uzui H., Yue H., Wang J., Toyoda K., Geshi T., Ueda T. (2006). Effects of folic acid and magnesium on the production of homocysteine-induced extracellular matrix metalloproteinase-2 in cultured rat vascular smooth muscle cells. Circ. J..

[84] Mohtashamian A., Soleimani A., Gilasi H.R., Kheiripour N., Taba S.M.M., Sharifi N. (2023). Association of Zinc Status with Matrix Metalloproteinases, Advanced Glycation End-Products, and Blood Pressure in Patients with Chronic Kidney Disease. Biol. Trace Elem. Res..

[85] Xu C., Huang Z., Liu L., Luo C., Lu G., Li Q., Gao X. (2015). Zinc Regulates Lipid Metabolism and MMPs Expression in Lipid Disturbance Rabbits. Biol. Trace Elem. Res..

[86] Siméon A., Monier F., Emonard H., Gillery P., Birembaut P., Hornebeck W., Maquart F.X. (1999). Expression and activation of matrix metalloproteinases in wounds: Modulation by the tripeptide-copper complex glycyl-L-histidyl-L-lysine-Cu^2+^. J. Investig. Dermatol..

[87] Varzaneh M.B., Rahmani H., Jahanian R., Mahdavi A.H., Perreau C., Perrot G., Brézillon S., Maquart F.-X. (2016). Effects of Dietary Copper-Methionine on Matrix Metalloproteinase-2 in the Lungs of Cold-Stressed Broilers as an Animal Model for Pulmonary Hypertension. Biol. Trace Elem. Res..

[88] Upagupta C., Shimbori C., Alsilmi R., Kolb M. (2018). Matrix abnormalities in pulmonary fibrosis. Eur. Respir. Rev..

[89] Lee C.-M., Park J.W., Cho W.-K., Zhou Y., Han B., Yoon P.O., Chae J., Elias J.A., Lee C.G. (2014). Modifiers of TGF-beta1 effector function as novel therapeutic targets of pulmonary fibrosis. Korean J. Intern. Med..

[90] Bellocq A., Azoulay E., Marullo S., Flahault A., Fouqueray B., Philippe C., Cadranel J., Baud L. (1999). Reactive oxygen and nitrogen intermediates increase transforming growth factor-beta1 release from human epithelial alveolar cells through two different mechanisms. Am. J. Respir. Cell Mol. Biol..

[91] Khalil N., O’Connor R.N., Unruh H.W., Warren P.W., Flanders K.C., Kemp A., Bereznay O.H., Greenberg A.H. (1991). Increased production and immunohistochemical localization of transforming growth factor-beta in idiopathic pulmonary fibrosis. Am. J. Respir. Cell Mol. Biol..

[92] Raghow B., Irish P., Kang A.H. (1989). Coordinate regulation of transforming growth factor beta gene expression and cell proliferation in hamster lungs undergoing bleomycin-induced pulmonary fibrosis. J. Clin. Investig..

[93] Coker R.K., Laurent G.J., Shahzeidi S., Lympany P.A., du Bois R.M., Jeffery P.K., McAnulty R.J. (1997). Transforming growth factors-beta 1, -beta 2, and -beta 3 stimulate fibroblast procollagen production in vitro but are differentially expressed during bleomycin-induced lung fibrosis. Am. J. Pathol..

[94] Conte E., Gili E., Fagone E., Fruciano M., Iemmolo M., Vancheri C. (2014). Effect of pirfenidone on proliferation, TGF-beta-induced myofibroblast differentiation and fibrogenic activity of primary human lung fibroblasts. Eur. J. Pharm. Sci..

[95] Choi J.Y., Kang M., Jung J.H., Kim W.J., Yang H.S., Lee K., Lee J., Yang S.-R., Rhee C.K., Hong S.H. (2024). Exposure of lung fibroblasts to PM_2.5_ and lead (Pb) induces fibrosis and apoptosis in alveolar epithelial cells via a paracrine effect. Ecotoxicol. Environ. Saf..

[96] Shivakumar K., Kumar B.P. (1997). Magnesium deficiency enhances oxidative stress and collagen synthesis in vivo in the aorta of rats. Int. J. Biochem. Cell Biol..

[97] Zeitlmayr S., Zierler S., Staab-Weijnitz C.A., Dietrich A., Geiger F., Horgen F.D., Gudermann T., Breit A. (2022). TRPM7 restrains plasmin activity and promotes transforming growth factor-β1 signaling in primary human lung fibroblasts. Arch. Toxicol..

[98] Gu L., Zhu Y.J., Yang X., Guo Z.J., Xu W.B., Tian X.L. (2007). Effect of TGF-beta/Smad signaling pathway on lung myofibroblast differentiation. Acta Pharmacol. Sin..

[99] Yang Q., Zhang P., Liu T., Zhang X., Pan X., Cen Y., Liu Y., Zhang H., Chen X. (2019). Magnesium isoglycyrrhizinate ameliorates radiation-induced pulmonary fibrosis by inhibiting fibroblast differentiation via the p38MAPK/Akt/Nox4 pathway. Biomed. Pharmacother..

[100] Luo X., Deng Q., Xue Y., Zhang T., Wu Z., Peng H., Xuan L., Pan G. (2021). Anti-Fibrosis Effects of Magnesium Lithospermate B in Experimental Pulmonary Fibrosis: By Inhibiting TGF-βRI/Smad Signaling. Molecules.

[101] Anttinen H., Oikarinen A., Puistola U., Pääkkö P., Ryhänen L. (1985). Prevention by zinc of rat lung collagen accumulation in carbon tetrachloride injury. Am. Rev. Respir. Dis..

[102] Elwej A., Ghorbel I., Chaabane M., Chelly S., Boudawara T., Zeghal N. (2024). Mitigating effects of selenium and zinc on oxidative stress and biochemical and histopathological changes in lung during prenatal and lactational exposure rats to barium chloride. Environ. Sci. Pollut. Res. Int..

[103] Tian B., Patrikeev I., Ochoa L., Vargas G., Belanger K.K., Litvinov J., Boldogh I., Ameredes B.T., Motamedi M., Brasier A.R. (2017). NF-κB Mediates Mesenchymal Transition, Remodeling, and Pulmonary Fibrosis in Response to Chronic Inflammation by Viral RNA Patterns. Am. J. Respir. Cell Mol. Biol..

[104] Wen X., Liu Y., Bai Y., Li M., Fu Q., Zheng Y. (2018). LOXL2, a copper-dependent monoamine oxidase, activates lung fibroblasts through the TGF-β/Smad pathway. Int. J. Mol. Med..

[105] Niu Y.-Y., Zhang Y.-Y., Zhu Z., Zhang X.-Q., Liu X., Zhu S.-Y., Song Y., Jin X., Lindholm B., Yu C. (2020). Elevated intracellular copper contributes a unique role to kidney fibrosis by lysyl oxidase mediated matrix crosslinking. Cell Death Dis..

[106] Chung K.W., Song S.H., Kim M.S. (2021). Synergistic effect of copper and amino acid mixtures on the production of extracellular matrix proteins in skin fibroblasts. Mol. Biol. Rep..

[107] Nguyen X.X., Nishimoto T., Takihara T., Mlakar L., Bradshaw A.D., Feghali-Bostwick C. (2021). Lysyl oxidase directly contributes to extracellular matrix production and fibrosis in systemic sclerosis. Am. J. Physiol. Lung Cell Mol. Physiol..

[108] Lee S., Lee D.-K., Jeon S., Kim S.-H., Jeong J., Kim J.S., Cho J.H., Park H., Cho W.-S. (2021). Combination effect of nanoparticles on the acute pulmonary inflammogenic potential: Additive effect and antagonistic effect. Nanotoxicology.

[109] Besiktepe N., Kayalar O., Ersen E., Oztay F. (2017). The copper dependent-lysyl oxidases contribute to the pathogenesis of pulmonary emphysema in chronic obstructive pulmonary disease patients. J. Trace Elem. Med. Biol..

[110] Marttila-Ichihara F., Elima K., Auvinen K., Veres T.Z., Rantakari P., Weston C., Miyasaka M., Adams D., Jalkanen S., Salmi M. (2017). Amine oxidase activity regulates the development of pulmonary fibrosis. FASEB J..

[111] Chaudhary N.I., Roth G.J., Hilberg F., Müller-Quernheim J., Prasse A., Zissel G., Schnapp A., Park J.E. (2007). Inhibition of PDGF, VEGF and FGF signalling attenuates fibrosis. Eur. Respir. J..

[112] Hilberg F., Roth G.J., Krssak M., Kautschitsch S., Sommergruber W., Tontsch-Grunt U., Garin-Chesa P., Bader G., Zoephel A., Quant J. (2008). BIBF 1120: Triple angiokinase inhibitor with sustained receptor blockade and good antitumor efficacy. Cancer Res..

[113] Chiou H.Y.C., Wang C.W., Chen S.C., Tsai M.L., Lin M.H., Hung C.H., Kuo C.H. (2023). Copper Exposure Induces Epithelial-Mesenchymal Transition-Related Fibrotic Change via Autophagy and Increase Risk of Lung Fibrosis in Human. Antioxidants.

[114] Skoczyńska A., Gruszczyński L., Wojakowska A., Ścieszka M., Turczyn B., Schmidt E. (2016). Association between the Type of Workplace and Lung Function in Copper Miners. BioMed Res. Int..

[115] Pohanka M. (2019). Copper and copper nanoparticles toxicity and their impact on basic functions in the body. Bratisl. Lek. Listy.

[116] Waas W.F., Dalby K.N. (2003). Physiological concentrations of divalent magnesium ion activate the serine/threonine specific protein kinase ERK2. Biochemistry.

[117] Barbagallo M., Dominguez L.J. (2007). Magnesium metabolism in type 2 diabetes mellitus, metabolic syndrome and insulin resistance. Arch. Biochem. Biophys..

[118] Iyer A.K.V., Ramesh V., Castro C.A., Kaushik V., Kulkarni Y.M., Wright C.A., Venkatadri R., Rojanasakul Y., Azad N. (2015). Nitric oxide mediates bleomycin-induced angiogenesis and pulmonary fibrosis via regulation of VEGF. J. Cell Biochem..

[119] Fehrenbach H., Kasper M., Haase M., Schuh D., Muller M. (1999). Differential immunolocalization of VEGF in rat and human adult lung, and in experimental rat lung fibrosis: Light, fluorescence, and electron microscopy. Anat. Rec..

[120] Cameli P., Bergantini L., Salvini M., Refini R.M., Pieroni M., Bargagli E., Sestini S. (2019). Alveolar concentration of nitric oxide as a prognostic biomarker in idiopathic pulmonary fibrosis. Nitric Oxide.

[121] Xu F., Wang Q., Jiang L., Zhu F., Yang L., Zhang S. (2022). Evaluation of Nitric Oxide Fluctuation via a Fast, Responsive Fluorescent Probe in Idiopathic Pulmonary Fibrosis Cells and Mice Models. Anal. Chem..

[122] Rock E., Astier C., Lab C., Malpuech C., Nowacki W., Gueux E., Mazur A., Rayssiguier Y. (1995). Magnesium deficiency in rats induces a rise in plasma nitric oxide. Magnes. Res..

[123] Gomez N.N., Davicino R.C., Biaggio V.S., Bianco G.A., Alvarez S.M., Fischer P., Masnatta L., Rabinovich G.A., Gimenez M.S. (2006). Overexpression of inducible nitric oxide synthase and cyclooxygenase-2 in rat zinc-deficient lung: Involvement of a NF-κB dependent pathway. Nitric Oxide.

[124] Cuzzocrea S., Persichini T., Dugo L., Colasanti M., Musci G. (2003). Copper induces type II nitric oxide synthase in vivo. Free Radic. Biol. Med..

[125] Murray P.J. (2017). Macrophage polarization. Annu. Rev. Physiol..

[126] Lech M., Anders H.-J. (2013). Macrophages and fibrosis: How resident and infiltrating mononuclear phagocytes orchestrate all phases of tissue injury and repair. Biochim. Biophys. Acta.

[127] Song E., Ouyang N., Hörbelt M., Antus B., Wang M., Exton M.S. (2000). Influence of alternatively and classically activated macrophages on fibrogenic activities of human fibroblasts. Cell. Immunol..

[128] Luo Q., Deng D., Li Y., Shi H., Zhao J., Qian Q., Wang W., Cai J., Yu W., Liu J. (2023). TREM2 Insufficiency Protects against Pulmonary Fibrosis by Inhibiting M2 Macrophage Polarization. Int. Immunopharmacol..

[129] Zhang M., Zhang J., Hu H., Zhou Y., Lin Z.W., Jing H., Sun B. (2024). Multiomic analysis of monocyte-derived alveolar macrophages in idiopathic pulmonary fibrosis. J. Transl. Med..

[130] Yokoyama T., Oono H., Miyamoto A., Ishiguro S., Nishio A. (2003). Magnesium-deficient medium enhances NO production in alveolar macrophages isolated from rats. Life Sci..

[131] Lang C.J., Hansen M., Roscioli E., Jones J., Murgia C., Ackland M.L., Zalewski P., Anderson G., Ruffin R. (2011). Dietary zinc mediates inflammation and protects against wasting and metabolic derangement caused by sustained cigarette smoke exposure in mice. Biometals.

[132] Abul H.T., Abul A.T., Al-Athary E.A., Behbehani A.E., Khadadah M.E., Dashti H.M. (1995). Interleukin-1α (IL-1α) production by alveolar macrophages in patients with acute lung diseases: The influence of zinc supplementation. Mol. Cell. Biochem..

[133] Hodgkinson V., Petris M.J. (2012). Copper homeostasis at the host-pathogen interface. J. Biol. Chem..

[134] Kim H.W., Chan Q., Afton S.E., Caruso J.A., Lai B., Weintraub N.L., Qin Z. (2012). Human macrophage ATP7A is localized in the trans-Golgi apparatus, controls intracellular copper levels, and mediates macrophage responses to dermal wounds. Inflammation.

[135] Oyarzún G.M.J., Sánchez S.A., Dussaubat D.N., Miller A.M.E., González B.S. (2017). Effect of copper sulphate on the lung damage induced by chronic intermittent exposure to ozone. Rev. Med. Chil..

[136] Knoell D.L., Smith D.A., Sapkota M., Heires A.J., Hanson C.K., Smith L.M., Poole J.A., Wyatt T.A., Romberger D.J. (2019). Insufficient zinc intake enhances lung inflammation in response to agricultural organic dust exposure. J. Nutr. Biochem..

[137] Joshi P.C., Mehta A., Jabber W.J., Fan X., Guidot D.M. (2009). Zinc deficiency mediates alcohol-induced alveolar epithelial and macrophage dysfunction in rats. Am. J. Respir. Cell Mol. Biol..

[138] Kim K.K., Dotson M.R., Agarwal M., Yang J., Bradley P.B., Subbotina N., Osterholzer J.J., Sisson T.H. (2018). Efferocytosis of apoptotic alveolar epithelial cells is sufficient to initiate lung fibrosis. Cell Death Dis..

[139] Sisson T.H., Mendez M., Choi K., Subbotina N., Courey A., Cunningham A., Dave A., Engelhardt J.F., Liu X., White E.S. (2010). Targeted injury of type II alveolar epithelial cells induces pulmonary fibrosis. Am. J. Respir. Crit. Care Med..

[140] Barkauskas C.E., Cronce M.J., Rackley C.R., Bowie E.J., Keene D.R., Stripp B.R., Randell S.H., Noble P.W., Hogan B.L.M. (2013). Type 2 alveolar cells are stem cells in adult lung. J. Clin. Investig..

[141] Parimon T., Yao C., Stripp B.R., Noble P.W., Chen P. (2020). Alveolar Epithelial Type II Cells as Drivers of Lung Fibrosis in Idiopathic Pulmonary Fibrosis. Int. J. Mol. Sci..

[142] Selman M., King T.E., Pardo A. (2001). Idiopathic pulmonary fibrosis: Prevailing and evolving hypotheses about its pathogenesis and implications for therapy. Ann. Intern. Med..

[143] Alder J.K., Barkauskas C.E., Limjunyawong N., Stanley S.E., Kembou F., Tuder R.M., Hogan B.L.M., Mitzner W., Armanios M. (2015). Telomere dysfunction causes alveolar stem cell failure. Proc. Natl. Acad. Sci. USA.

[144] Snetselaar R., van Batenburg A.A., van Oosterhout M.F.M., Kazemier K.M., Roothaan S.M., Peeters T., Van Der Vis J.J., Goldschmeding R., Grutters J.C., Van Moorsel C.H.M. (2017). Short telomere length in IPF lung associates with fibrotic lesions and predicts survival. PLoS ONE.

[145] Barbas-Filho J.V., Ferreira M.A., Sesso A., Kairalla R.A., Carvalho C.R., Capelozzi V.L. (2001). Evidence of type II pneumocyte apoptosis in the pathogenesis of idiopathic pulmonary fibrosis (IFP)/usual interstitial pneumonia (UIP). J. Clin. Pathol..

[146] Li X., Wang Y., Liang J., Bi Z., Ruan H., Cui Y., Ma L., Wei Y., Zhou B., Zhang L. (2021). Bergenin attenuates bleomycin-induced pulmonary fibrosis in mice via inhibiting TGF-β1 signaling pathway. Phytother. Res..

[147] Chu L., Zhuo J., Huang H., Chen W., Zhong W., Zhang J., Meng X., Zou F., Cai S., Zou M. (2024). Tetrandrine alleviates pulmonary fibrosis by inhibiting alveolar epithelial cell senescence through PINK1/Parkin-mediated mitophagy. Eur. J. Pharmacol..

[148] Xiao H., Peng L., Jiang D., Liu Y., Zhu L., Li Z., Geng J., Xie B., Huang X., Wang J. (2022). IL-17A promotes lung fibrosis through impairing mitochondrial homeostasis in type II alveolar epithelial cells. J. Cell. Mol. Med..

[149] Kasper M., Barth K. (2017). Potential contribution of alveolar epithelial type I cells to pulmonary fibrosis. Biosci. Rep..

[150] Shi X., Zhu L., Wang S., Zhu W., Li Q., Wei J., Feng D., Liu M., Chen Y., Sun X. (2022). Magnesium Hydride Ameliorates Endotoxin-Induced Acute Respiratory Distress Syndrome by Inhibiting Inflammation, Oxidative Stress, and Cell Apoptosis. Oxidative Med. Cell Longev..

[151] Liang J., Huang G., Liu X., Taghavifar F., Liu N., Wang Y., Deng N., Yao C., Xie T., Kulur V. (2022). 1ZIP8/SIRT1 axis regulates alveolar progenitor cell renewal in aging and idiopathic pulmonary fibrosis. J. Clin. Investig..

[152] Moschini E., Gualtieri M., Colombo M., Fascio U., Camatini M., Mantecca P. (2013). The modality of cell-particle interactions drives the toxicity of nanosized CuO and TiO_2_ in human alveolar epithelial cells. Toxicol. Lett..

[153] Isler J.A., Skalet A.H., Alwine J.C. (2005). Human cytomegalovirus infection activates and regulates the unfolded protein response. J. Virol..

[154] Tang Y.-W., Johnson J.E., Browning P.J., Cruz-Gervis R.A., Davis A., Graham B.S., Brigham K.L., Oates J.A., Loyd J.E., Stecenko A.A. (2003). Herpesvirus DNA is consistently detected in lungs of patients with idiopathic pulmonary fibrosis. J. Clin. Microbiol..

[155] Geng J., Liu Y.Y., Dai H., Wang C. (2022). Fatty Acid Metabolism and Idiopathic *Pulmonary fibrosis*. Front. Physiol..

[156] Chu S.G., Villalba J.A., Liang X., Xiong K., Tsoyi K., Ith B., Ayaub E.A., Tatituri R.V., Byers D.E., Hsu F.-F. (2019). Palmitic acid-rich high-fat diet exacerbates experimental pulmonary fibrosis by modulating endoplasmic reticulum stress. Am. J. Respir. Cell Mol. Biol..

[157] Kim H., Yoo H.J., Lee K.M., Song H.E., Kim S.J., Lee J.O., Hwang J.J., Song J.W. (2021). Stearic acid attenuates profibrotic signalling in idiopathic pulmonary fibrosis. Respirology.

[158] Soma M., Cunnane S.C., Horrobin D.F., Manku M.S., Honda M., Hatano M. (1988). Effects of low magnesium diet on the vascular prostaglandin and fatty acid metabolism in rats. Prostaglandins.

[159] Dieck H.T., Döring F., Fuchs D., Roth H.P., Daniel H. (2005). Transcriptome and proteome analysis identifies the pathways that increase hepatic lipid accumulation in zinc-deficient rats. J. Nutr..

[160] Zhu D., Zhang Y., Li S., Gan L., Feng H., Nie W. (2014). Enteral omega-3 fatty acid supplementation in adult patients with acute respiratory distress syndrome: A systematic review of randomized controlled trials with meta-analysis and trial sequential analysis. Intensive Care Med..

[161] Charbeneau R.P., Peters-Golden M. (2005). Eicosanoids: Mediators and therapeutic targets in fibrotic lung disease. Clin. Sci..

[162] Silva R.C., Landgraf M.A., Hiyane M.I., Pacheco-Silva A., Câmara N.O., Landgraf R.G. (2010). Leukotrienes produced in allergic lung inflammation activate alveolar macrophages. Cell. Physiol. Biochem..

[163] Wetterholm A., Macchia L., Haeggström J.Z. (1994). Zinc and other divalent cations inhibit purified leukotriene A4 hydrolase and leukotriene B4 biosynthesis in human polymorphonuclear leukocytes. Arch. Biochem. Biophys..

[164] Gales C., Stoica B., Rusu-Zota G., Nechifor M. (2024). Montelukast Influence on Lung in Experimental Diabetes. Medicina.

[165] Jenkins K.J., Kramer J.K. (1992). Changes in lipid composition of calf tissues by excess dietary zinc. J. Dairy Sci..

[166] Nechifor M., Cuciureanu M., Chelarescu D., Ciubotariu D., Pascu M. (2008). Magnesium and other bivalent cations influence upon sodium montelukast effect in experimental-induced thermoalgesia. Magnes. Res..

[167] Genovese T., Cuzzocrea S., Di Paola R., Mazzon E., Mastruzzo C., Catalano P., Sortino M., Crimi N., Caputi A.P., Thiemermann C. (2005). Effect of rosiglitazone and 15-deoxy-Delta12, 14-prostaglandin J2 on bleomycin-induced lung injury. Eur. Respir. J..

[168] Oga T., Matsuoka T., Yao C., Nonomura K., Kitaoka S., Sakata D., Kita Y., Tanizawa K., Taguchi Y., Chin K. (2009). Prostaglandin F(2α) receptor signaling facilitates bleomycin-induced pulmonary fibrosis independently of transforming growth factor-beta. Nat. Med..

[169] Brennan E.P., Cacace A., Godson C. (2017). Specialized pro-resolving mediators in renal fibrosis. Mol. Asp. Med..

[170] Mariqueo T.A., Zúñiga-Hernández J. (2020). Omega-3 derivatives, specialized pro-resolving mediators: Promising therapeutic tools for the treatment of pain in chronic liver disease. Prostaglandins Leukot. Essent. Fat. Acids.

[171] Serhan C.N., Dalli J., Karamnov S., Choi A., Park C.-K., Xu Z.-Z., Ji R.-R., Zhu M., Petasis N.A. (2012). Macrophage proresolving mediator maresin 1 stimulates tissue regeneration and controls pain. FASEB J..

[172] Xie C., Li X., Wu J., Liang Z., Deng F., Xie W., Zhu M., Zhu J., Zhu W., Geng S. (2015). Anti-inflammatory Activity of Magnesium Isoglycyrrhizinate Through Inhibition of Phospholipase A2/Arachidonic Acid Pathway. Inflammation.

[173] Ludwig P., Petrich K., Schewe T., Diezel W. (1995). Inhibition of eicosanoid formation in human polymorphonuclear leukocytes by high concentrations of magnesium ions. Biol. Chem. Hoppe Seyler.

[174] Mezna M., Ahmad T., Chettibi S., Drainas D., Lawrence A.J. (1994). Zinc and barium inhibit the phospholipase A2 from Naja naja atra by different mechanisms. Biochem. J..

[175] Nito C., Kamada H., Endo H., Niizuma K., Myer D.J., Chan P.H. (2008). Role of the p38 mitogen-activated protein kinase/cytosolic phospholipase A2 signaling pathway in blood-brain barrier disruption after focal cerebral ischemia and reperfusion. J. Cereb. Blood Flow. Metab..

[176] Milanino R., Passarella E., Velo G.P. (1978). Adjuvant arthritis in young copper-deficient rats. Agents Actions.

[177] Ponist S., Valentová J., Bezáková L., Oblozinský M. (2006). Antilipoxygenase activity of copper complexes of aminoalkanoate type. Neuro Endocrinol. Lett..

[178] Li X., Xie M., Lu C., Mao J., Cao Y., Yang Y., Wei Y., Liu X., Cao S., Song Y. (2020). Design and synthesis of Leukotriene A4 hydrolase inhibitors to alleviate idiopathic pulmonary fibrosis and acute lung injury. Eur. J. Med. Chem..

[179] Khodabakhsh P., Khoie N., Dehpour A.R., Abdollahi A., Ghazi-Khansari M., Shafaroodi H. (2022). Montelukast suppresses the development of irritable bowel syndrome phenotype possibly through modulating NF-κB signaling in an experimental model. Inflammopharmacology.

[180] Fireman E., Schwartz Y., Mann A., Greif J. (2004). Effect of montelukast, a cysteinyl receptor antagonist, on myofibroblasts in interstitial lung disease. J. Clin. Immunol..

[181] Shimbori C., Shiota N., Okunishi H. (2011). Effects of montelukast, a cysteinyl-leukotriene type 1 receptor antagonist, on the pathogenesis of bleomycin-induced pulmonary fibrosis in mice. Eur. J. Pharmacol..

[182] Ong T.J., Kemp P.J., Oliver R.E., McArdle H.J. (1995). Characterization of zinc uptake and its regulation by arachidonic acid in fetal type II pneumocytes. Am. J. Physiol..

[183] Xu Y., Mizuno T., Sridharan A., Du Y., Guo M., Tang J., Wikenheiser-Brokamp K.A., Perl A.-K.T., Funari V.A., Gokey J.J. (2016). Single-cell RNA sequencing identifies diverse roles of epithelial cells in idiopathic pulmonary fibrosis. JCI Insight.

[184] Zhang K., Wang L., Hong X., Chen H., Shi Y., Liu Y., Liu J., Liu J.P. (2021). Pulmonary Alveolar Stem Cell Senescence, Apoptosis, and Differentiation by p53-Dependent and -Independent Mechanisms in Telomerase-Deficient Mice. Cells.

[185] Takahashi T., Munakata M., Ohtsuka Y., Nisihara H., Nasuhara Y., Kamachi-Satoh A., Dosaka-Akita H., Homma Y., Kawakami Y. (2002). Expression and alteration of ras and p53 proteins in patients with lung carcinoma accompanied by idiopathic pulmonary fibrosis. Cancer.

[186] Shetty S., Idell S. (2023). Caveolin-1-Related Intervention for Fibrotic Lung Diseases. Cells.

[187] Iezhitsa I., Lambuk L., Agarwal R., Agarwal P., Peresypkina A., Pobeda A., Ismail N.M. (2021). Magnesium acetyltaurate prevents retinal damage and visual impairment in rats through suppression of NMDA-induced upregulation of NF-κB, p53 and AP-1 (c-Jun/c-Fos). Neural Regen. Res..

[188] Chen P.M., Wu T.C., Wang Y.C., Cheng Y.C., Sheu G.T., Chen C.Y., Lee H. (2013). Activation of NF-κB by SOD2 promotes the aggressiveness of lung adenocarcinoma by modulating NKX2-1-mediated IKKβ expression. Carcinogenesis.

[189] Inchehsablagh B.R., Soufi F.G., Koochakkhani S., Azarkish F., Farshidi H., Eslami M., Mahmoodi M., Soltani N., Eftekhar E. (2023). Magnesium Supplementation Affects the Expression of Sirtuin1, Tumor Protein P53 and Endothelial Nitric Oxide Synthase Genes in Patients with Atherosclerosis: A Double-Blind, Randomized, Placebo-Controlled Trial. Indian J. Clin. Biochem..

[190] Clegg M.S., Hanna L.A., Niles B.J., Momma T.Y., Keen C.L. (2005). Zinc deficiency-induced cell death. UBMB Life.

[191] Formigari A., Gregianin E., Irato P. (2013). The effect of zinc and the role of p53 in copper-induced cellular stress responses. J. Appl. Toxicol..

[192] Sauve A.A., Schramm V.L. (2004). SIR2: The biochemical mechanism of NAD(+)-dependent protein deacetylation and ADP-ribosyl enzyme intermediates. Curr. Med. Chem..

[193] Zhang Y., Huang W., Zheng Z., Wang W., Yuan Y., Hong Q., Lin J., Li X., Meng Y. (2021). Cigarette smoke-inactivated SIRT1 promotes autophagy-dependent senescence of alveolar epithelial type 2 cells to induce pulmonary fibrosis. Free Radic. Biol. Med..

[194] Peng Z., Zhang W., Qiao J., He B. (2018). Melatonin attenuates airway inflammation via SIRT1 dependent inhibition of NLRP3 inflammasome and IL-1β in rats with COPD. Int. Immunopharmacol..

[195] Nogueiras R., Habegger K.M., Chaudhary N., Finan B., Banks A.S., Dietrich M.O., Horvath T.L., Sinclair D.A., Pfluger P.T., Tschöop M.H. (2012). Sirtuin 1 and sirtuin 3: Physiological modulators of metabolism. Physiol. Rev..

[196] Kobayashi E.H., Suzuki T., Funayama R., Nagashima T., Hayashi M., Sekine H., Tanaka N., Moriguchi T., Motohashi H., Nakayama K. (2016). Nrf2 suppresses macrophage inflammatory response by blocking proinflammatory cytokine transcription. Nat. Commun..

[197] Foster P.S., Tay H.L., Oliver B.G. (2022). Deficiency in the zinc transporter ZIP8 impairs epithelia renewal and enhances lung fibrosis. J. Clin. Investig..

[198] Jablonski R.P., Kim S., Cheresh P., Williams D.B., Morales-Nebreda L., Cheng Y., Yeldandi A., Bhorade S., Pardo A., Selman M. (2017). SIRT3 deficiency promotes lung fibrosis by augmenting alveolar epithelial cell mitochondrial DNA damage and apoptosis. FASEB J..

[199] Ravishankar S., Ashraf Q.M., Fritz K., Mishra O.P., Delivoria-Papadopoulos M. (2001). Expression of Bax and Bcl-2 proteins during hypoxia in cerebral cortical neuronal nuclei of newborn piglets: Effect of administration of magnesium sulfate. Brain Res..

[200] Wei Z., Sun X., He Q., Zhao Y., Wu Y., Han X., Wu Z., Chu X., Guan S. (2022). Nephroprotective effect of magnesium isoglycyrrhizinate against arsenic trioxide-induced acute kidney damage in mice. Exp. Ther. Med..

[201] Larson-Casey J.L., Deshane J.S., Ryan A.J., Thannickal V.J., Carter A.B. (2016). Macrophage Akt1 kinase-mediated mitophagy modulates apoptosis resistance and pulmonary fibrosis. Immunity.

[202] Wu Q., Zhang K.-J., Jiang S.-M., Fu L., Shi Y., Tan R.-B., Cui J., Zhou Y. (2020). p53: A Key Protein That Regulates Pulmonary Fibrosis. Oxidative Med. Cell Longev..

[203] Komiya C., Tanaka M., Tsuchiya K., Shimazu N., Mori K., Furuke S., Miyachi Y., Shiba K., Yamaguchi S., Ikeda K. (2017). Antifibrotic effect of pirfenidone in a mouse model of human nonalcoholic steatohepatitis. Sci. Rep..

[204] Cho H.-J., Hwang J.-A., Yang E.J., Kim E.-C., Kim J.-R., Kim S.Y., Kim Y.Z., Park S.C., Lee Y.-S. (2022). Nintedanib induces senolytic effect via STAT3 inhibition. Cell Death Dis..

[205] Zischka H., Kroemer G. (2020). Copper—A novel stimulator of autophagy. Cell Stress.

[206] Xue Q., Kang R., Klionsky D.J. (2023). Copper metabolism in cell death and autophagy. Autophagy.

[207] Jian Z., Guo H., Liu H., Cui H., Fang J., Zuo Z., Deng J., Li Y., Wang X., Zhao L. (2020). Oxidative stress, apoptosis and inflammatory responses involved in copper-induced pulmonary toxicity in mice. Aging.

[208] Kang Z., Qiao N., Liu G., Chen H., Tang Z., Li Y. (2019). Copper-induced apoptosis and autophagy through oxidative stress-mediated mitochondrial dysfunction in male germ cells. Toxicol. Vitr..

[209] Derseh H.B., Perera K.U.E., Dewage S.N.V., Stent A., Koumoundouros E., Organ L., Pagel C.N., Snibson K.J. (2021). Tetrathiomolybdate Treatment Attenuates Bleomycin-Induced Angiogenesis and Lung Pathology in a Sheep Model of Pulmonary Fibrosis. Front. Pharmacol..

[210] Plataki M., Koutsopoulos A.V., Darivianaki A.V., Delides G., Siafakas N.M., Bouros D. (2005). Expression of apoptotic and antiapoptotic markers in epithelial cells in idiopathic pulmonary fibrosis. Chest.

[211] Ronan N., Bennett D.M., Khan K.A., McCarthy Y., Dahly D., Bourke L., Chelliah A., Cavazza A., O’rEgan K., Moloney F. (2018). Tissue and Bronchoalveolar Lavage Biomarkers in Idiopathic Pulmonary Fibrosis Patients on Pirfenidone. Lung.

[212] Xaubet A., Marin-Arguedas A., Lario S., Ancochea J., Morell F., Ruiz-Manzano J., Rodriguez-Becerra E., Rodriguez-Arias J.M., Iñigo P., Sanz S. (2003). Transforming growth factor-β1 gene polymorphisms are associated with disease progression in idiopathic pulmonary fibrosis. Am. J. Respir. Crit. Care Med..

[213] Wang H., Xu H., Lyu W., Xu Q., Fan S., Chen H., Wang D., Chen J., Dai J. (2022). KLF4 regulates TERT expression in alveolar epithelial cells in pulmonary fibrosis. Cell Death Dis..

[214] Demedts M., Behr J., Buhl R., Costabel U., Dekhuijzen R., Jansen H.M., MacNee W., Thomeer M., Wallaert B., Laurent F. (2005). High-dose acetylcysteine in idiopathic pulmonary fibrosis. N. Engl. J. Med..

[215] Raghu G., Selman M. (2015). Nintedanib and pirfenidone. New antifibrotic treatments indicated for idiopathic pulmonary fibrosis offer hopes and raises questions. Am. J. Respir. Crit. Care Med..

[216] Liu Y., Lu F., Kang L., Wang Z., Wang Y. (2017). Pirfenidone attenuates bleomycin-induced pulmonary fibrosis in mice by regulating Nrf2/Bach1 equilibrium. BMC Pulm. Med..

[217] Kou N., Chen Y.-B., Li X.-W., Xu D., Wang Y., Dong X.-R., Cui Y.-L., Wang Q. (2024). Pulmonary administration of tetrandrine loaded Zinc-Alginate nanogels attenuates pulmonary fibrosis in rats. Int. J. Pharm..

[218] Homma S., Azuma A., Taniguchi H., Ogura T., Mochiduki Y., Sugiyama Y., Nakata K., Yoshimura K., Takeuchi M., Kudoh S. (2012). Efficacy of inhaled N-acetylcysteine monotherapy in patients with early stage idiopathic pulmonary fibrosis. Respirology.

[219] Misra H.P., Rabideau C. (2000). Pirfenidone inhibits NADPH-dependent microsomal lipid peroxidation and scavenges hydroxyl radicals. Mol. Cell. Biochem..

[220] Leenders N.H.J., van Ittersum F.J., Hoekstra T., Hoenderop J.G.J., Vervloet M.G. (2018). Routine hemodialysis induces a decline in plasma magnesium concentration in most patients: A prospective observational cohort study. Sci. Rep..

[221] Rodelo-Haad C., Pendón-Ruiz de Mier M.V., Díaz-Tocados J.M., Martin-Malo A., Santamaria R., Muñoz-Castañeda J.R., Rodriguez M. (2020). The Role of Disturbed Mg Homeostasis in Chronic Kidney Disease Comorbidities. Front. Cell Dev. Biol..

[222] Qu C., Xu Y., Yang X., Lu X. (2020). Magnesium lithospermate B improves pulmonary artery banding induced right ventricular dysfunction by alleviating inflammation via p38MAPK pathway. Pulm. Pharmacol. Ther..

[223] Dos Santos M.I.S., Molle R.D., Silva F.M., Rodrigues T.W., Feldmann M., Forte G.C., Marostica P.J.C. (2020). Antioxidant Micronutrients and Essential Fatty Acids Supplementation on Cystic Fibrosis Outcomes: A Systematic Review. J. Acad. Nutr. Diet..

[224] Wang D., Zhu Z.-L., Lin D.-C., Zheng S.-Y., Chuang K.-H., Gui L.-X., Yao R.-H., Zhu W.-J., Sham J.S., Lin M.-J. (2021). Magnesium Supplementation Attenuates Pulmonary Hypertension via Regulation of Magnesium Transporters. Hypertension.

[225] Bethou A. (2021). Utility of Nebulized Magnesium Sulfate Therapy for Persistent Pulmonary Hypertension of Newborn. Indian J. Pediatr..

[226] Chen W., Chen A., Lian G., Yan Y., Liu J., Wu J., Gao G., Xie L. (2024). Zinc attenuates monocrotaline-induced pulmonary hypertension in rats through upregulation of A20. J. Mol. Cell. Cardiol..

[227] Li T., Luo X.-J., Wang E.-L., Li N.-S., Zhang X.-J., Song F.-L., Yang J.-F., Liu B., Peng J. (2019). Magnesium lithospermate B prevents phenotypic transformation of pulmonary arteries in rats with hypoxic pulmonary hypertension through suppression of NADPH oxidase. Eur. J. Pharmacol..

[228] Zhao D., Chen P., Chen M., Chen L., Wang L. (2024). Association of Magnesium Depletion Score with Congestive Heart Failure: Results from the NHANES 2007–2016. Biol. Trace Elem. Res..

[229] Lin B., Alexander R., Fritzen R., Mills S., Stewart A.J., McCowan C. (2025). Abnormal Plasma/Serum Magnesium, Copper, and Zinc Concentrations Associate with the Future Development of Cardiovascular Diseases. Nutrients.

[230] Wang W., Wang X., Luo J., Chen X., Ma K., He H., Li W., Cui J. (2021). Serum Copper Level and the Copper-to-Zinc Ratio Could Be Useful in the Prediction of Lung Cancer and Its Prognosis: A Case-Control Study in Northeast China. Nutr. Cancer.

[231] Feng Y., Gao M., Xu X., Liu H., Lu K., Song Z., Yu J., Liu X., Han X., Li L. (2024). Elevated serum magnesium levels prompt favourable outcomes in cancer patients treated with immune checkpoint blockers. Eur. J. Cancer.

[232] Charoenngam N., Ponvilawan B., Ungprasert P. (2021). Higher zinc intake is associated with decreased risk of lung cancer. J. Evid. Based Med..

